# Research Progress in Chitin/Chitosan-Based Biomass Adhesives: Extraction Processes, Composite and Chemical Modification

**DOI:** 10.3390/polym18030337

**Published:** 2026-01-27

**Authors:** Yizhang Luo, Ziying Zhang, Jiachen Zuo, Libo Zhang

**Affiliations:** 1 School of Materials Science and Engineering, Shenyang University of Chemical Technology, Shenyang 110142, China; 2313050424@stu.syuct.edu.cn; 2 Guangdong Provincial Engineering & Technology Center for Corrosion and Safety in Petrochemical Industry, School of Chemical Engineering, Guangdong University of Petrochemical Technology, Maoming 525000, China; zhangziying@gdupt.edu.cn (Z.Z.); zjcenormous@gdupt.edu.cn (J.Z.); 3 College of Engineering, China University of Petroleum-Beijing at Karamay, Karamay 834000, China

**Keywords:** chitin/chitosan, biomass-based adhesives, composite modification, chemical modification, biomineralization, industrialization challenge

## Abstract

Traditional fossil-based adhesives, hindered by issues such as formaldehyde emission, dependence on fossil resources, and poor biodegradability, struggle to meet the global demand for low-carbon green development. Biomass-based adhesives have thus emerged as a core alternative. Among them, chitin/chitosan derived from biomass waste such as shrimp and crab shells demonstrates significant potential in the adhesive field due to its renewability, controllable structure, biocompatibility, and inherent antibacterial properties. However, mainstream biomass adhesives like soy protein and starch adhesives suffer from poor water resistance and insufficient wet adhesion strength. Pure chitin/chitosan-based adhesive systems also exhibit low wet strength retention. Furthermore, the overall development faces challenges including high extraction costs, insufficient performance synergy, poor industrial compatibility, and a lack of standardized systems. This review follows the framework of “resource–extraction–modification–performance–application–challenges” to systematically summarize relevant research progress. It clarifies the molecular structure and intrinsic advantages of chitin/chitosan, outlines extraction processes such as acid/alkali and enzymatic methods, and characterization techniques including FT-IR and XRD. The review focuses on analyzing modification strategies such as composite modification, chemical modification, biomineralization, and biomimetic design, and verifies the application potential of these adhesives in wood processing, biomedicine, paper-based packaging, and other fields. Research indicates that chitin/chitosan-based adhesives provide an effective pathway for the green transformation of the adhesive industry. Future efforts should concentrate on developing green extraction processes, designing multifunctional integrated systems, and achieving full resource utilization of biomass. Additionally, establishing comprehensive standardized systems and promoting the translation of laboratory research into industrial applications are crucial to driving the industry’s green transition.

## 1. Introduction

Traditional fossil-based adhesives (e.g., urea–formaldehyde resin, phenolic resin) have long dominated fields such as wood processing and composite materials due to their cost advantages. However, their environmental drawbacks are increasingly prominent. Firstly, the issue of formaldehyde emissions persists: aldehyde-containing adhesives like urea–formaldehyde and melamine–formaldehyde resins continuously release formaldehyde during production, use, and aging. Long-term exposure can induce respiratory diseases and even carcinogenic risks, severely limiting their application in sensitive scenarios such as interior decoration and children’s furniture [1]. Secondly, their reliance on fossil resources is a concern. Sourced from non-renewable petroleum and coal, the stability and sustainability of their supply chains face severe challenges amidst the global energy crisis and rising extraction costs. Furthermore, it is difficult for these adhesives to biodegrade after disposal, leading to long-term environmental pollution, which contradicts global low-carbon goals and the green development concept [2]. Consequently, promoting the green transition of the adhesive industry has become an inevitable trend.

Biomass-based adhesives, characterized by their renewability, low pollution, and biodegradability, have emerged as a core direction for replacing traditional fossil-based adhesives. At the policy level, initiatives such as the EU’s Circular Economy Action Plan and China’s “14th Five-Year Plan” for the Raw Materials Industry explicitly advocate for the industrialization of biomass materials and encourage the development of zero-formaldehyde, low-carbon footprint green adhesive products [3]. From a market perspective, the global green adhesive market reached USD 12 billion in 2023 and is projected to grow at a compound annual growth rate (CAGR) of 8.5% by 2030, with biomass-based adhesives accounting for over 40% of this market. This reflects the industry’s urgent demand for environmentally friendly adhesives [4]. However, existing mainstream biomass adhesives (e.g., soybean protein adhesive, starch-based adhesive) commonly suffer from poor water resistance and insufficient wet-state bonding strength, making it difficult to meet the demands of complex application scenarios. There is a pressing need to develop novel biomass adhesive systems with superior performance [1,3].

**Figure 1:**
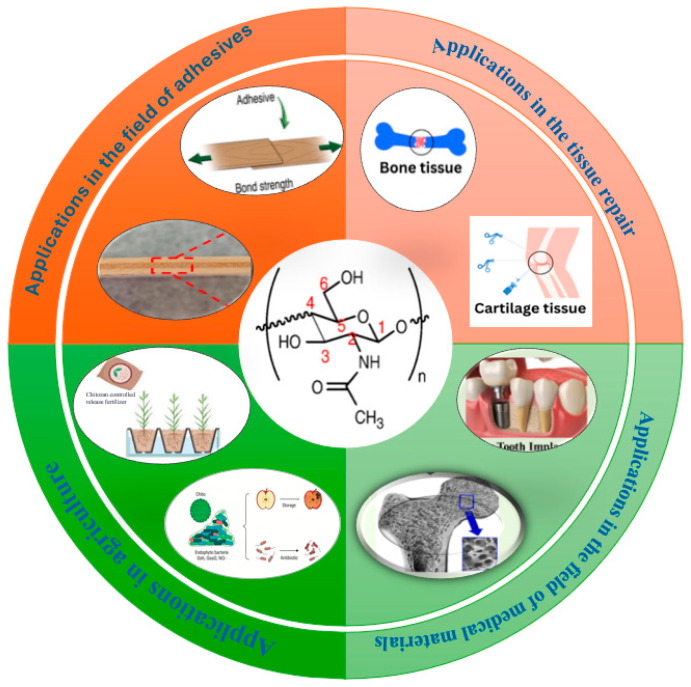
Exemplary Applications of Chitin and Chitosan in Multiple Fields. The picture is from the online color atlas.

Chitin/chitosan-based biomass adhesives demonstrate application potential across multiple fields due to their tunable properties and green characteristics, as shown in Figure 1.

In the field of wood and wood-based composites, they can be used for bonding plywood, particleboard, fiberboard, etc. For instance, plywood prepared using a composite adhesive of 3% chitosan and 10% corn starch (with citric acid as a crosslinker) achieves a dry internal bond (IB) strength of 0.7 MPa, with a 24 h wet-state IB retention rate of 45%. Although the dry strength is slightly lower than that of urea–formaldehyde resin (IB > 1.0 MPa), its formaldehyde emission is nearly zero. The cost is approximately 1.5–2 times that of urea–formaldehyde resin. Current industrial applications are concentrated in high-end, low-formaldehyde panels (e.g., particleboard for children’s furniture). However, challenges remain, including insufficient water resistance (wet strength often fails to meet load-bearing requirements) and poor adaptability to large-scale production processes (e.g., viscosity control is difficult to match with existing glue application equipment) [1,3].

In the biomedical field, they are suitable for applications such as dental bonding, tissue engineering scaffolds, and wound dressings. For example, genipin-crosslinked chitosan adhesive exhibits a shear strength of 1.8 MPa on dental implants with a 90% survival rate for L929 cells. Hydroxyapatite-mineralized chitosan adhesive can promote osteoblast adhesion. Catechol-grafted chitosan adhesive for medical dressings achieves an underwater wet-state adhesion strength of 1.2 MPa on skin and can gradually degrade into glucose units within the body (degradation period of 2–4 weeks) without toxic residue [5,6,7,8].

In other fields, they can be used for bonding paper and packaging materials (e.g., nanocellulose-composited chitosan adhesive achieves a dry bonding strength of 0.6 MPa on paper, suitable for flexible packaging), bonding bio-based/flame-retardant composites (e.g., phosphorylated chitosan adhesive with a Limiting Oxygen Index (LOI) of 28% used for flame-retardant wood composite panels), and also serve as functional coatings for environmental remediation (e.g., chitosan–graphene composite adhesive coated on soil particles to enhance aggregation and prevent water and soil erosion) [9,10,11,12].

Chitin/chitosan, as a highly promising biomass raw material, demonstrates irreplaceable advantages in the field of adhesives. Firstly, its resource advantages are significant. It is primarily derived from marine biomass waste such as shrimp shells, crab shells, and lobster shells (global annual waste exceeding 10 million tons), as well as terrestrial biological resources like cicada sloughs and fungal cell walls. This enables a “waste-to-resource conversion,” reducing raw material costs while alleviating environmental pressure. Secondly, chitin/chitosan exhibits strong controllability in structure and performance. Chitin (a β-(1,4)-2-acetamido-D-glucose polymer) can be converted into chitosan (containing abundant active amino and hydroxyl groups) through deacetylation. By adjusting the degree of deacetylation (DD), molecular weight, and chemical modifications, the adhesive strength, water resistance, and antibacterial properties of the adhesive can be precisely optimized. Thirdly, its functional diversity is outstanding, combining excellent biocompatibility with inherent antibacterial properties. It can not only be used for wood bonding but also extended to biomedical fields such as dental restoration and tissue engineering. Furthermore, it can inhibit wood mold growth and extend the service life of composite materials [13,14]. In recent years, breakthroughs in modification technologies, such as catechol grafting (mimicking mussel adhesion mechanisms) and nanocomposite formation (incorporating chitin nanocrystals for reinforcement) [5,11,15], have further enhanced the comprehensive performance of chitin/chitosan-based adhesives, advancing their transition from laboratory research toward industrial application.

Research on chitin/chitosan-based biomass adhesives began in the late 20th century, evolving through three stages: “basic exploration–composite modification–fine functionalization.” Early work focused on the performance regulation of pure chitosan adhesives, revealing their fundamental bonding potential, yet they are limited by poor water resistance. In the early 21st century, their preliminary application in wood bonding was advanced through compositing with starch, soybean protein, etc., achieving an internal bond (IB) strength exceeding 0.5 MPa [1]. Over the past decade, breakthroughs in chemical modification (e.g., catechol grafting, phosphorylation) and nanocomposite techniques (e.g., chitin nanocrystals, graphene) have increased wet-state bonding strength by 30–50%, raised the limiting oxygen index (LOI) above 28%, and expanded applications to biomedical fields such as dental restoration [10,12,14,15]. Current research emphasizes performance optimization (e.g., citric acid crosslinking reduces 24 h water absorption to below 20% [9,16]) and scenario adaptation (e.g., antibacterial adhesives achieve mold inhibition rates over 80% [13]). However, significant shortcomings remain, including high raw material extraction costs (green enzymatic methods cost 2–3 times more than traditional ones [17,18]), inadequate performance synergy (single modifications struggle to balance strength and antibacterial properties [13,19]), and poor industrial compatibility (small-batch preparation mismatches existing equipment [9,13,16,20,21]. Following a framework of “resources–preparation–modification–performance–application–challenges,” this review systematically covers chitin/chitosan extraction processes, adhesive preparation (pure systems/composites/chemical modification), performance optimization (water resistance/strength/antibacterial properties), multi-scenario applications, and industrial bottlenecks, aiming to consolidate research progress and clarify future directions [1,2,3,22].

This review focuses on chitin/chitosan-based biomass adhesives and follows a logical framework centered on “green transition demand–technological breakthroughs–industrial implementation.” It begins by addressing the shortcomings of traditional fossil-based adhesives, including formaldehyde emissions, resource dependence, and poor degradability. Drawing on policy and market data, it highlights the necessity of developing biomass adhesives and analyzes the limitations of current mainstream products, thereby emphasizing the unique advantages of chitin/chitosan in terms of renewable resources, controllable structure, and functional diversity. Subsequently, the molecular structural characteristics of chitin/chitosan are analyzed, such as the high crystallinity of chitin and the abundant active functional groups in chitosan, alongside their inherent properties, including biocompatibility, degradability, and antibacterial activity, thereby establishing the theoretical foundation of the review.

Following this, the article adopts a progressive structure of “foundation–optimization–innovation” to systematically elaborate on the performance limitations of pure systems and the performance improvements achieved through composite modification (integration with natural/synthetic polymers and nanofillers), chemical modification (crosslinking, grafting, functionalization), as well as biomineralization and bioinspired design. These advancements are contextualized within application scenarios such as wood processing, biomedicine, and paper packaging to validate their practical value.

Finally, the review confronts existing bottlenecks, including high costs, insufficient performance synergy, and lack of standards, proposing future directions such as green processes, multifunctional design, and full resource utilization. This forms a complete research cycle, providing a comprehensive reference for both academic research and industrial development in this field.

## 2. Fundamental Characteristics of Chitin/Chitosan

**Figure 2:**
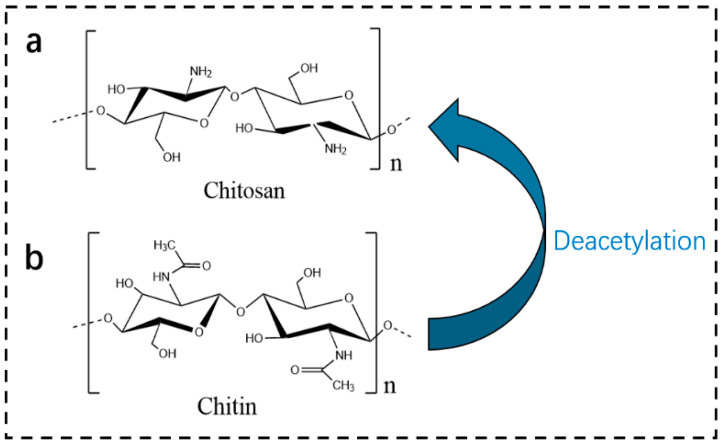
Structural formulas of chitin and chitosan. (**a**) Chitosan and (**b**) chitin. The picture is from the online color atlas.

Chitin is a linear polysaccharide formed by β-(1,4)-linked N-acetyl-D-glucosamine units connected via glycosidic bonds. Within its molecular chains, abundant acetyl amino (-NHCOCH_3_) and hydroxyl (-OH) groups form ordered crystalline structures through hydrogen bonding, endowing it with high mechanical strength and chemical stability [23,24,25]. The structural formulas of chitin and chitosan are shown in Figure 2.

Chitosan is a derivative obtained through the deacetylation of chitin, a process that removes acetyl amino groups. Its molecular chain primarily consists of β-(1,4)-linked D-glucosamine units, while retaining some acetyl amino groups, with a degree of deacetylation (DD) typically ranging from 50% to 100%. The introduction of amino groups (-NH_2_) enhances the polarity of the molecular chains. Furthermore, these amino groups can be protonated to form -NH_3_^+^ under acidic conditions, resulting in a positively charged linear structure. This characteristic provides a crucial foundation for subsequent chemical reactions and interfacial interactions [26,27]. X-ray diffraction (XRD) analysis reveals that the crystallinity of chitin (approximately 70–80%) is significantly higher than that of chitosan (approximately 40–60%), which is attributed to the more regular hydrogen-bonding network in chitin molecules [28].

As adhesive materials, chitin and chitosan exhibit favorable biocompatibility, enabling effective integration with biological tissues such as wood cells and human cells without significant toxicity or irritation. This makes them suitable for applications in wood bonding as well as biomedical fields, including dental restoration and tissue engineering. For instance, chitosan-based dental adhesives have demonstrated gingival cell viability rates exceeding 90% [14]. Concurrently, chitin and chitosan are biodegradable. Under the action of enzymes (e.g., chitosanase) or microorganisms, their molecular chains can be progressively degraded into glucose units, ultimately assimilated by the environment or metabolized, thereby avoiding waste pollution and aligning with the requirements for green materials [25,29].

Furthermore, chitin and chitosan possess abundant reactive sites. The hydroxyl groups, amino groups (in chitosan), and acetamido groups (in chitin) within their molecular chains can participate in various reactions such as crosslinking, grafting, and coordination. For example, amino groups can form Schiff base crosslinks with aldehydes (e.g., glutaraldehyde [30,31], dialdehyde starch), while hydroxyl groups can undergo esterification with acid anhydrides (e.g., maleic anhydride), providing a chemical foundation for tailoring adhesive properties [13,24,32]. The cross-linking treatment of chitosan with glutaraldehyde leads to significant changes in its structure and morphology, primarily manifested as reduced crystallinity and altered surface topography. X-ray diffraction (XRD) analysis reveals that pure chitosan exhibits sharp diffraction peaks at 2θ = 10° and 20°, indicating its high crystallinity, which originates from strong hydrogen bonds formed between hydroxyl and amino groups as well as the ordered arrangement of molecular chains. In contrast, the chitosan–glutaraldehyde copolymer (CLCG) after cross-linking shows the disappearance of these characteristic peaks, with only a broad and weak diffraction peak appearing near 2θ = 15°. This indicates that the cross-linking reaction disrupts the hydrogen-bond network among chitosan molecules, replaces part of the hydroxyl and amino groups, and results in disordered chain arrangement, forming a material dominated by an amorphous structure [33]. Moreover, scanning electron microscopy (SEM) observations demonstrate that the surface morphology changes from smooth and compact to rough and porous after cross-linking, further confirming the alteration of its microstructure due to the cross-linking reaction [34]. These structural changes, particularly the decrease in crystallinity and the increase in porosity, contribute to enhancing the specific surface area and ion accessibility of the material, thereby improving its charge storage and transport performance in electrochemical applications such as supercapacitors [33,35].

Additionally, these adhesives possess inherent antibacterial properties. The protonated amino groups in chitosan can disrupt microbial cell membranes, inhibiting the growth of bacteria (e.g., Escherichia coli, Staphylococcus aureus) and fungi (e.g., wood mold fungi), with antibacterial rates reaching 80–95%. This capability contributes to extending the service life of wood-based composite materials [13,36].

As biomass adhesives, chitin and chitosan demonstrate significant inherent advantages. Firstly, they exhibit considerable interfacial bonding potential. The polar functional groups (hydroxyl and amino groups) within their molecular chains can form strong hydrogen bonds with hydroxyl groups on substrate surfaces, such as wood. Simultaneously, the positively charged amino groups of chitosan can generate electrostatic attraction with negatively charged sites on the substrate surface, enhancing interfacial adhesion. Pure chitosan adhesive can achieve a dry-state tensile shear strength of 1.0–1.5 MPa on wood [36,37].

Secondly, chitin and chitosan offer excellent functional extensibility. Owing to their abundant reactive sites, functionalities such as antibacterial and flame-retardant properties can be introduced through compounding or modification. Examples include compounding with phosphate esters to impart flame retardancy or grafting with catechol groups to enhance wet-state adhesion, all without requiring additional functional additives [5,12,15].

Finally, they are resource-efficient and environmentally friendly. Sourced from biomass waste, their production process can achieve low pollution levels, and the materials are biodegradable after disposal, aligning with the principles of a “circular economy” [38,39,40].

## 3. Preparation and Purification Technologies of Chitin/Chitosan

### 3.1. Source of Raw Materials

Chitin/chitosan is widely sourced from marine and terrestrial biomass resources, primarily in the form of waste materials, enabling the resource recycling of “transforming waste into valuable resources.” Its main sources and characteristics are summarized in Table 1 below.

Based on the aforementioned raw material properties, the differences in content, purity, and structure of chitin/chitosan from various sources directly determine their suitable application scenarios in adhesives. For instance, chitin/chitosan derived from shrimp and crab shells, due to its low cost and high yield, is more suitable for the large-scale production of general-purpose wood adhesives. In the study by Boussetta [1], the chitosan used to prepare a corn starch–mimosa tannin composite adhesive originated from shrimp shell processing waste. Its degree of deacetylation was controlled between 70% and 80%, enabling the formation of a stable cross-linked network with starch and tannin via hydrogen bonds, thereby enhancing the internal bond strength of the adhesive. High-molecular-weight chitin from lobster shells, after nano-processing (e.g., preparation of chitin nanocrystals), can serve as a reinforcing filler. In the research by Liu [11], nanocrystals prepared from lobster shell chitin, when added at 5%, increased the elastic modulus of the adhesive by over 200 MPa. In contrast, chitin/chitosan with low impurity content from sources like cicada sloughs and fungi holds greater advantages in biomedical adhesive applications such as dental bonding and wound dressings. The chitosan used by Zhang et al. [14] for dental bonding was extracted from cicada sloughs, with an ash content below 0.5% and a gingival cell survival rate of 92%, meeting biocompatibility requirements.

Furthermore, the regional distribution characteristics of raw materials also influence the selection of extraction processes. Coastal regions tend to utilize marine waste such as shrimp and crab shells, achieving large-scale extraction through established acid–base methods. For instance, a coastal plant mentioned in the study by Saeedi [50] employs a process involving demineralization with 10% hydrochloric acid and deproteinization with 5% sodium hydroxide to obtain chitin with a purity exceeding 80% from shrimp and crab shells. Inland regions, on the other hand, can leverage fungal fermentation technology to procure raw materials locally, thereby reducing transportation costs. As introduced in the study by Krake [51], an inland bio-factory can stably produce fungal biomass containing 10% to 12% chitin through submerged fermentation using Aspergillus niger, with fermentation conditions being easily adjustable. This “locally adapted” model of raw material utilization provides diversified resource assurance for the industrialization of chitin/chitosan-based biomass adhesives.

### 3.2. Extraction Process

The extraction process of chitin/chitosan directly determines its purity, structure, and subsequent adhesive performance. Conventional methods, primarily acid–base treatments, are well-established but face issues related to pollution and energy consumption. In recent years, green extraction technologies—such as enzymatic hydrolysis and microbial fermentation—have advanced rapidly due to their environmental benefits. Meanwhile, purification and refining processes focus on the removal of impurities and the regulation of key properties, such as the degree of deacetylation and molecular weight, to provide customized raw materials for adhesive applications. The different extraction methods and their characteristics are summarized in Table 2.

From the perspective of process compatibility, the traditional acid–base method remains the mainstream choice for chitin/chitosan used in wood adhesives. In Boussetta’s study [1] on the preparation of the CSMT (corn starch–mimosa tannin–chitosan) adhesive, chitosan was obtained via deacetylation with 30% NaOH (80 °C, 4 h), with the degree of deacetylation (DD) controlled between 75% and 80%. This allows the chitosan to form hydrogen bonds with the hydroxyl groups of starch and the phenolic hydroxyl groups of tannin, resulting in an internal bond (IB) strength of 0.45 MPa for the particleboard. However, the acidic and alkaline wastewater generated by this process requires treatment, limiting its environmental sustainability.

Among green extraction technologies, the enzymatic method is more suitable for high-value-added adhesives. For the chitosan used in dental bonding, Zhang et al. [14] employed alkaline protease (enzyme activity 3000 U/g) for deproteinization (40 °C, 6 h), achieving a protein removal rate of 88% and a chitosan molecular weight retention rate exceeding 90%. This process resulted in a gingival cell survival rate of 92%, meeting biocompatibility requirements. In contrast, ultrasound-assisted acid–base treatment is suitable for the upgrading of conventional factories. For instance, as mentioned in the research by Nessa et al. [41], ultrasound assistance (300 W, 30 min) during demineralization with 10% hydrochloric acid reduced the demineralization time from 4 h to 1.5 h and decreased acid consumption by 30%, while achieving a chitin purity of 86%. This method is applicable to the large-scale production of raw materials for wood adhesives.

In purification and refining processes, the regulation of the degree of deacetylation (DD) and molecular weight directly influences adhesive performance. For example, in the study by Cai et al. [9], chitosan with a DD of 60% prepared via stepwise deacetylation formed a denser hydrogen-bond network when cross-linked with citric acid, reducing the 24 h water absorption of the adhesive to 18%, which is significantly better than the 28% observed for chitosan with a high DD (90%). In the research by Todorović et al. [20], chitosan with a molecular weight of 5 × 10^5^ Da was obtained through ethanol fractionation (50% concentration). After grafting with polyvinyl acetate, its viscosity was controlled within the range of 5000–8000 mPa·s, making it suitable for plywood gluing equipment. This effectively avoided the issues of insufficient viscosity (<2000 mPa·s) caused by low-molecular-weight chitosan (1 × 10^5^ Da) or gelation problems associated with high-molecular-weight chitosan (1 × 10^6^ Da). Furthermore, impurity removal is crucial for medical adhesives. In the experiment by Sajomsang et al. [47], chitin extracted from cicada sloughs was decolorized with activated carbon (3%, 50 °C, 2 h), reducing the ash content from 3.2% to 0.8%. When used to prepare dental adhesives, no significant cytotoxicity was observed, whereas untreated samples led to a decrease in cell viability to 75%.

Traditional acid–base methods and green extraction technologies exhibit significant divergence in core performance and application suitability. In terms of cost and efficiency, the extraction cost per ton of traditional acid–base methods is only one-half to one-third that of enzymatic hydrolysis, with a processing time reduced by more than 50%. For example, demineralization of shrimp and crab shells takes just 1–6 h using traditional methods, compared to 4–12 h for enzymatic hydrolysis [70]. This makes traditional methods the mainstream choice for large-scale production of raw materials for wood adhesives.

However, green technologies offer notable environmental advantages. Enzymatic hydrolysis generates no acid or alkaline wastewater, while microbial fermentation can achieve product purity exceeding 90%, significantly higher than the approximately 80% typical of traditional methods [23]. These features make green technologies more suitable for high-end applications such as biomedicine, where impurity residues are a critical concern.

Regarding product performance, the high-temperature and strong alkali conditions of traditional acid–base methods can lead to degradation of chitin/chitosan molecular chains, with molecular weight loss rates reaching 20–30%, which may subsequently weaken adhesive cohesion [12]. In contrast, enzymatic hydrolysis preserves over 90% of the molecular weight, and microbial fermentation allows more precise control of the degree of deacetylation (DD) with a deviation of only ±2% [3,67]. This provides a superior raw material foundation for the molecular design of high-performance adhesives.

From the perspective of industrial feasibility, traditional acid–base methods benefit from mature equipment and stable processes, but require costly wastewater treatment facilities, accounting for 30–40% of total production costs [71]. Among green technologies, ultrasonic/microwave-assisted methods can reduce acid and alkali consumption in traditional processes by 20–40%, making them suitable for technological upgrades in existing plants. Meanwhile, microbial fermentation remains largely confined to laboratory-scale or small-batch production due to its long cycle time (24–72 h) and low yield [72].

### 3.3. Structure and Performance Characterization

**Figure 3:**
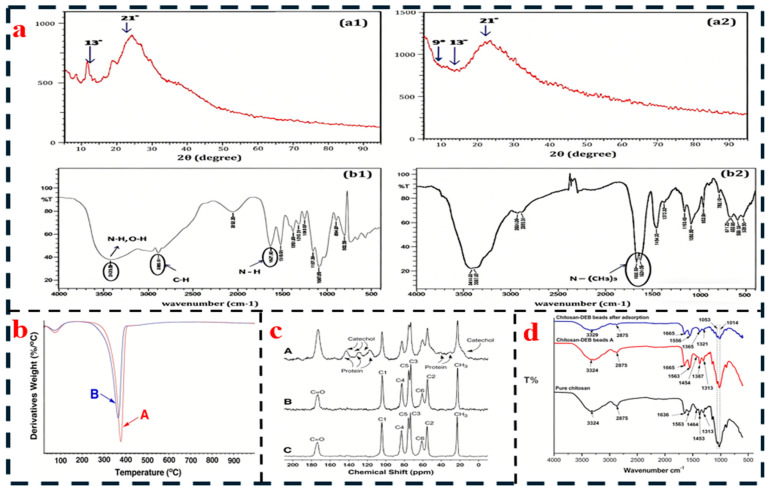
Typical Characterization Techniques for Chitin/Chitosan. (**a**). Comparison of XRD and FTIR results for (**a1**,**b1**) pristine chitosan and (**a2**,**b2**) N,N,N-trimethyl chitosan prepared using a DES-based solvent. (**b**). Differential thermal analysis (DTA) curves of chitin from paddy crab shells (A) and chitin from cicada sloughs (B). (**c**). 13C CP/MAS NMR spectra: cicada sloughs (A), chitin from cicada sloughs (B), and chitin from paddy crab shells (C). (**d**). Fourier-transform infrared (FTIR) spectra of pure chitosan microspheres and chitosan-DES microspheres before and after malachite green adsorption [9,13,33,73].

The structural and performance characterization of chitin/chitosan serves as a critical link bridging extraction processes and adhesive applications. It is essential to elucidate molecular chain characteristics (e.g., functional groups, degree of deacetylation) through chemical structural analysis and to assess macroscopic application potential (e.g., viscosity, thermal stability) via physical performance testing. Concurrently, analyzing the influence of extraction processes on these properties provides a basis for the selection of raw materials and the formulation design of adhesives. Typical characterization techniques for chitin/chitosan are summarized in Figure 3.

The chemical structure characterization of chitin/chitosan, which is critical for assessing their compatibility with other adhesive components such as cross-linkers and fillers, focuses on precisely determining the functional group composition, structural regularity, and purity of the molecular chains. Fourier-transform infrared spectroscopy (FT-IR) provides a qualitative assessment of the conversion from chitin to chitosan by monitoring characteristic peak intensities; for example, the gradual weakening of the acetyl C=O stretching vibration at ~1650 cm^−1^ alongside the strengthening of the amino N-H bending vibration at ~1600 cm^−1^, where at 80% deacetylation (DD) the latter’s peak area can be 2.3 times that of the former, offering a rapid means to verify suitability for reactions with cross-linking agents such as dialdehyde starch or citric acid [74,75]. Proton nuclear magnetic resonance (^1^H NMR) enables precise quantitative control of DD, typically within ±2% error, by integrating the acetyl -CH_3_ peak at δ = 2.05 ppm relative to the sugar ring hydrogen peaks (δ = 3.0–4.0 ppm); this technique was employed to adjust chitosan DD to 75–80% for a corn starch–mimosa tannin adhesive, ensuring stable hydrogen bonding that yielded a particleboard internal bond strength of 0.45 MPa [1]. X-ray diffraction (XRD) reveals the influence of crystalline regularity on properties: native chitin exhibits high crystallinity (70–80%) with characteristic peaks at 2θ = 9.5°, 19.5°, and 23.5°, imparting mechanical strength but poor solubility, whereas deacetylated chitosan shows reduced crystallinity (40–60%) and improved solubility—a balance adjustable for coating needs, as demonstrated when enzymatically treated cicada-sourced chitin showed a crystallinity drop from 72% to 65% alongside a 30% solubility increase [47,76]. Finally, elemental analysis (EA) serves as a key purity verification tool, where nitrogen content (theoretically 6.89% for fully deacetylated chitosan) correlates with DD and indicates residual impurities; for instance, optimized desalting of shrimp-shell chitosan achieved a nitrogen content of 6.2% (≈90% DD) with Cl residue < 0.1%, meeting stringent biosafety requirements for medical adhesives [45,77].

Physical performance testing focuses on the macroscopic application characteristics of chitin/chitosan, requiring evaluation based on the entire “coating-curing-service life” cycle of adhesives. Viscosity, as a core indicator of coatability, directly determines the application method. A 2% aqueous chitosan solution (dissolved in 1% acetic acid) with a viscosity below 2000 mPa·s at 25 °C is suitable for spray coating in thin paper bonding. If the viscosity falls within the range of 2000–8000 mPa·s, it is appropriate for roller coating in plywood production, while exceeding 10,000 mPa·s can lead to uneven coating, necessitating optimization through graft modification or molecular weight adjustment. In the study by Todorović et al. [20], grafting chitosan with polyvinyl acetate increased its viscosity from 3000 mPa·s to 6500 mPa·s, effectively avoiding issues such as excessively thin adhesive layers caused by low viscosity and gelation problems from high viscosity, thereby perfectly meeting the requirements of plywood roller coating.

Thermal stability is evaluated through thermogravimetric analysis (TGA). The initial decomposition temperature (T_5_%) of chitin is approximately 280 °C, while that of chitosan decreases to around 250 °C due to the presence of amino groups. This parameter must align with the hot-pressing temperature in wood processing (typically 160–180 °C) to prevent material degradation from affecting bond strength. Phosphorylation modification can increase the T_5_% of chitosan to 290 °C, meeting the high-temperature hot-pressing requirements of particleboard while imparting flame retardancy to the adhesive (limiting oxygen index, LOI > 28%) [12,32].

Crystallinity and solubility jointly determine the cohesion and homogeneity of the adhesive. Chitosan with a crystallinity of 50–60% exhibits adequate cohesion (dry-state shear strength > 1.0 MPa) while being readily soluble in dilute acids to form a homogeneous adhesive phase. For instance, fungal-derived chitosan with a crystallinity of 45% achieves a solubility of 95% in 1% acetic acid, significantly outperforming shrimp shell-derived chitosan (crystallinity 55%, solubility 82%). This enables the preparation of high-solid-content (15%) adhesives, avoiding interfacial bonding defects caused by precipitation [78].

### 3.4. Performance Testing Framework for Chitin-Based Biopolymer Adhesives

The performance evaluation system for chitin-based biopolymer adhesives encompasses substrate selection, curing protocols, and multi-dimensional performance validation. The substrates are primarily poplar veneers, with common dimensions including 120 mm × 120 mm × 1.5 mm, 400 mm × 400 mm × 1.5 mm, and 22 cm × 12 cm × 2 mm. Some studies employ beech veneers (100 mm × 20 mm × 1.5 mm) [31]. Curing parameters vary significantly depending on the adhesive type. For instance, CS-PAA@Ca^2+^ systems, hemicellulose–chitosan composites, and SM/DCS composite adhesives are typically cured at 120 °C for 2.5–6.5 min under a pressure of 1–1.67 MPa [79]. The optimal curing condition for CSC-G carboxylated chitosan–glucose adhesive is 160 °C for 3 min at 1 MPa [79]. In contrast, chitosan–dopamine composite adhesives utilize a flexible curing scheme, ranging from room temperature to 120 °C for 2 h, adjusted as needed [80]. For tests without explicit standard protocols, industry-accepted custom methods are employed. Bond strength evaluation is conducted under multiple scenarios. Following Chinese standards, tests include dry strength at room temperature, wet strength after immersion in water at 63 ± 3 °C for 3 h, and boiling water strength after immersion in boiling water at 93 ± 3 °C for 3 h [80]. A rigorous custom test employs a “4+4+1” cyclic procedure: immersion in boiling water for 4 h → drying for 20 h → re-immersion in boiling water for 4 h → immersion in cold water for 1 h [45]. European standard tensile shear tests are performed with a 20 mm × 20 mm overlap area under controlled conditions of 23 ± 2 °C and 50 ± 5% relative humidity [81]. Thermal stability is characterized by Thermogravimetric Analysis (TGA) under a nitrogen atmosphere, heating from room temperature to 800 °C at a rate of 10 °C/min, and Differential Scanning Calorimetry (DSC), also under nitrogen, heating at 10 °C/min to record the curing exothermic peak [82]. Antifungal performance is assessed by continuous observation over 30 days under conditions of 30 °C and 99% relative humidity [83].

Aging resistance is validated through an accelerated aging cycle: immersion in hot water at 49 ± 2 °C for 1 h → steam treatment for 3 h → freezing at −12 ± 3 °C for 20 h → drying at 99 ± 2 °C for 3 h → steam treatment for 3 h → drying at 99 ± 2 °C for 18 h [84]. Water absorption/residual rate is determined by measuring weight changes after immersion in water at 63 °C for 12 h followed by drying to constant weight. The Limiting Oxygen Index (LOI), indicating the minimum oxygen concentration to support combustion, is tested following the UL-94 standard [85]. The performance testing system for chitin adhesives is shown in Table 3.

### 3.5. Correlation of Key Parameters with Bonding Performance in Chitosan-Based Adhesives

The dry/wet shear strength, water resistance, and thermal stability of chitosan-based adhesives are collectively determined by the degree of deacetylation, molecular weight, and crosslinking density through their influence on intermolecular interactions, interfacial bonding capacity, and network structural stability. The synergistic optimization of these three parameters is pivotal for enhancing the overall performance of the adhesive. The different extraction methods and their characteristics in Table 4.

Degree of Deacetylation (DD) is a core structural parameter of chitosan-based adhesives, representing the ratio of amino groups (-NH_2_) to acetamido groups (-NHCOCH_3_). In acidic conditions, amino groups protonate to form -NH_3_^+^, which enhances interfacial bonding with wood surfaces through electrostatic interactions with hydroxyl (-OH) and carboxyl (-COOH) groups, while also serving as reactive sites for crosslinking with agents such as citric acid and oxidized starch to form ester and amide bonds [91].

Data indicate that increasing the DD from 75% to 90% improves dry shear strength by an average of 40–60% [37]. The enhancement in wet shear strength is more pronounced, rising from <0.6 MPa to approximately 1.4 MPa, attributable to the denser crosslinked network formed at higher DD, which effectively impedes water penetration. However, when DD exceeds 95%, strong intermolecular hydrogen bonding increases crystallinity, reducing solubility and interfacial wettability, and leading to a slight decline in adhesive strength [10].

Molecular weight (Mw) influences adhesive performance by modulating viscosity, penetration, and cohesive strength. Low-molecular-weight chitosan (Mw < 50,000 Da) exhibits good solubility and high penetration, enabling mechanical interlocking within wood pores. However, short molecular chains result in weak cohesion, making the interface prone to debonding under stress, with dry shear strength generally below 1.5 MPa [76].

Medium-molecular-weight chitosan (Mw = 50,000–300,000 Da) balances penetration and cohesion. It penetrates micron-scale wood pores to form mechanical interlock while developing a strong cohesive network through chain entanglement and crosslinking, achieving dry shear strengths of 2.3–5.6 MPa [51]. High-molecular-weight chitosan (Mw > 500,000 Da) shows significantly increased viscosity (>50 Pa·s), leading to poor coating uniformity and limited penetration. This often results in thick adhesive layers at the wood interface, while excessive chain entanglement causes uneven crosslinking. Consequently, internal stress buildup under wet conditions can cause cracking, reducing wet shear strength by more than 30% [5].

Crosslinking density affects bonding performance by altering the stability of the network structure. Uncrosslinked or lightly crosslinked chitosan adhesives rely mainly on physical interactions, tending to swell and disintegrate after water immersion, with wet shear strength typically below 0.5 MPa [32]; rate crosslinking (e.g., using citric acid, TPP, or epoxy lignosulfonate) forms covalent networks, enhancing both cohesive strength and water resistance, yielding dry strength > 2.0 MPa and wet strength > 0.8 MPa [92].

However, excessively high crosslinking density (e.g., crosslinker content >20%) increases brittleness, preventing effective stress dissipation at the interface and leading to adhesive layer fracture under load, thereby reducing bond strength. Furthermore, crosslinking density exhibits synergistic effects with DD and Mw: high-DD chitosan requires moderate crosslinking to avoid excessive crystallinity; medium-Mw chitosan benefits from appropriate crosslinking to compensate for cohesive deficiency. Optimizing these three parameters jointly enables superior overall adhesive performance [70].

Based on the correlations among these parameters, the performance of chitosan adhesives can be optimized according to the following principles: select chitosan with DD = 85–90% to balance reactivity and solubility; control molecular weight within 100,000–200,000 Da to ensure both penetration and cohesion; and regulate crosslinking density using agents such as citric acid or oxidized starch to maintain a gel content of 60–80% [50]. In practical applications, for indoor plywood, priority may be given to increasing DD (e.g., 90%) to enhance dry strength; for humid environments (e.g., kitchen furniture), crosslinking density should be optimized (e.g., 10–15% crosslinker addition); for low-density woods (e.g., pine), lower-molecular-weight chitosan (Mw ≈ 80,000 Da) may be used to improve penetration. Future research may focus on graft modification (e.g., catechol functionalization) to further harmonize these parameters and improve bonding stability under extreme conditions [40].

### 3.6. Performance Comparison Between Composite-Modified Adhesive and the Original Adhesive

The composite modification strategy significantly enhances the core performance and practical application reliability of adhesives. The performance differences and repeated-use performance between the modified adhesives and their pristine counterparts can be compared across multiple dimensions.

Regarding bonding strength and water-resistant stability, pristine chitosan adhesives suffer from singular intermolecular forces and insufficient crosslinking density, resulting not only in limited dry shear strength (typically below 1.2 MPa) but also in susceptibility to interfacial debonding under humid conditions or repeated dry–wet cycles. Their wet shear strength often drops below 0.7 MPa, making them inadequate for the long-term requirements of materials like plywood [93]. In contrast, composite modification leads to a qualitative leap in performance. For instance, the composite of chitosan with epigallocatechin gallate (EGCG) achieves a dry shear strength of 1.98 MPa and maintains a wet shear strength of 1.17 MPa after immersion in hot water at 63 °C for 3 h. Even after 5 dry–wet cycles, the strength retention rate remains above 75%, far superior to the rapid deterioration characteristic of the pristine adhesive [94]. The carboxymethylated chitosan–glucose (CSC-G) adhesive, through the construction of a covalent crosslinked network, attains a wet shear strength of 0.71 MPa after boiling water immersion for 3 h After accelerated aging cycle tests, its bonding strength decreased by only 28%, demonstrating excellent stability under repeated-use conditions [17].

In terms of multifunctional integration and environmental adaptability, pristine adhesives are generally plagued by functional simplicity, susceptibility to mildew, and poor tolerance to extreme conditions. For example, soy protein-based adhesives are prone to mold growth during storage and repeated use, while pure chitosan adhesives lack flame-retardant properties, limiting their application in fields like construction and furniture [5]. Composite modification overcomes these deficiencies through the synergistic action of multiple components. The HBPE-CS adhesive combines the antibacterial properties of chitosan with the flame retardancy of biopolyester. It shows no mold growth after 28 days of storage at 20 °C and 60% relative humidity. Plywood bonded with this adhesive self-extinguishes within 76 s in an alcohol blowtorch test and maintains structural integrity after three burn–cool cycles, whereas plywood bonded with pristine chitosan adhesive is prone to complete combustion [58]. The chitosan–lignosulfonate composite adhesive (GL-C), through epoxidation modification, forms a dense three-dimensional network, increasing the contact angle from 62° for pristine chitosan to 68°, indicating enhanced hydrophobicity. In scenarios involving repeated contact with water media (e.g., adhesive bonding for exterior wall panels), the interfacial bonding stability is significantly superior to that of the pristine adhesive, which is susceptible to water absorption and swelling [21].

Concerning the scope of application and long-term reliability, pristine adhesives are often limited to bonding single material types and are prone to failure under repeated stress or environmental fluctuations. For example, pure poly(vinyl acetate) (PVAc) adhesive exhibits insufficient wet bonding strength (<1 MPa) for wood and is susceptible to cracking after repeated thermal cycles. Composite modification greatly enhances compatibility and durability. The chitosan-grafted-poly(vinyl acetate) (CS-g-PVAc) adhesive, by reducing the proportion of fossil-based components, achieves a dry shear strength of 6.9 MPa and a wet strength of 1.8 MPa, far exceeding the performance of pristine PVAc adhesive. After multiple disassembly–rebonding cycles, the bonding strength retention rate remains above 80%, making it suitable for repeated adjustments in wood processing [20]. The chitosan–chitin nanocrystals (ChNCs)–lysozyme composite adhesive, at a protein-to-ChNCs mass ratio of 1:20, exhibits a 25% increase in lap shear load compared to the pristine ChNCs adhesive. Under repeated shear stress, the synergistic formation of a supramolecular structure endows it with self-healing capability, resulting in a significantly slower rate of mechanical property degradation compared to single-component adhesives. These modification strategies, by constructing multiple crosslinked networks and introducing functionally synergistic components, not only address the core issues of pristine adhesives—such as low strength, poor water resistance, and functional simplicity—but also demonstrate stable performance output in repeated-use scenarios, providing a guarantee for their long-term application in practical engineering [95].

### 3.7. Main Application Fields of Chitosan and Its Chemically Modified Derivatives

Chitosan and its chemically modified derivatives exhibit broad application potential across multiple fields due to their favorable biocompatibility, biodegradability, and structural tunability. Their specific application scenarios are closely related to the modification methods employed.

In the food field, chitosan nanoparticles prepared via amino deprotonation or ionic cross-linking with tripolyphosphate (TPP) serve as efficient food-grade Pickering emulsifiers. They can stabilize oil-in-water emulsions containing high-value oils such as roasted coffee oil. Deprotonated chitosan nanoparticles (538–938 nm) enhance emulsion viscosity by forming a network structure in the continuous phase, while TPP-cross-linked nanoparticles (331–413 nm), due to their uniform size, confer superior emulsion stability. Both types of nanoparticles improve the bioaccessibility of oils and bioactive components, providing a novel approach for stabilizing active ingredients in food systems [96].

The wood bonding field represents a significant application direction for modified chitosan derivatives, with the core advantage of replacing formaldehyde-based adhesives to achieve environmentally friendly bonding. A single chitosan-based adhesive, through optimization of spread amount (1000 g·m^−2^), open assembly time (10 min), and hot-pressing parameters (55 °C/105 min), can achieve a dry shear strength of 2.82 MPa. Chitosan with high deacetylation degree and low molecular weight shows better wet strength, meeting the requirements of EN 204 standard D3 grade [89]. Composite modifications further expand their application scope: Citric acid (CA) cross-linking modification forms ester and amide bonds via supramolecular self-assembly, allowing viscosity tunability between 450 and 14,733 mPa·s to suit different bonding needs for plywood and particleboard. The dry/wet shear strengths reach 2.1 MPa and 1.1 MPa, respectively, complying with the GB/T 9846-2015 standard [9]. Sulfonated lignin modified with epichlorohydrin (GTE) and cross-linked with chitosan constructs a dense three-dimensional network, increasing the wet strength of plywood in 63 °C hot water to 0.84 MPa [21]. Adhesives modified with dynamic covalent bonds, incorporating borate esters and imine bonds, are not only recyclable (retaining 88% of original strength after recovery) but also exhibit excellent low-temperature flexibility (maintaining 3.63 MPa strength even at −196 °C) and mold resistance [90]. Furthermore, the complex formed between chitosan, polystyrene sulfonate (PSS), and kraft lignin (KL) through π-π stacking and hydrogen bonding achieves dry/wet shear strengths of 2.39 MPa and 1.40 MPa, respectively, meeting the ASTM D4690 standard. These modification strategies provide technical support for the industrialization of eco-friendly wood adhesives.

In the field of wastewater treatment, glutaraldehyde (Glu) cross-linked chitosan, by adjusting the Glu/chitosan monomer molar ratio to construct a porous network, demonstrates high-efficiency adsorption performance for organic pollutants such as p-nitrophenol (PNP) in water. Studies show optimal adsorption at a Glu/NH_2_ molar ratio of 4:1, with an adsorption capacity of 0.572 mmol/g under pH = 9.0 conditions. The adsorption mechanism is primarily electrostatic, as the binding capacity between phenolate anions and cationic sites on chitosan is significantly enhanced under alkaline conditions, offering a green and efficient adsorbent material for treating organic pollutant-containing wastewater [30].

Additionally, chitosan and its modified derivatives hold potential applications in biomedicine, packaging materials, and other fields. Chitosan oligosaccharide-based materials can serve as drug carriers and wound healing dressings, with their dynamic covalent networks offering both biocompatibility and degradability. Chitosan–lignin composite films, due to their good thermal stability and mechanical properties, can be developed into biodegradable food packaging materials. The synergistic effect between amino groups, borate, and phenolic hydroxyl groups endows them with excellent anti-mold and antibacterial properties, applicable for preservation treatments in wood, food, and related fields [90].

## 4. Preparation Technology of Chitin/Chitosan-Based Biomass Adhesives

### 4.1. Pure Chitosan Adhesive

**Figure 4:**
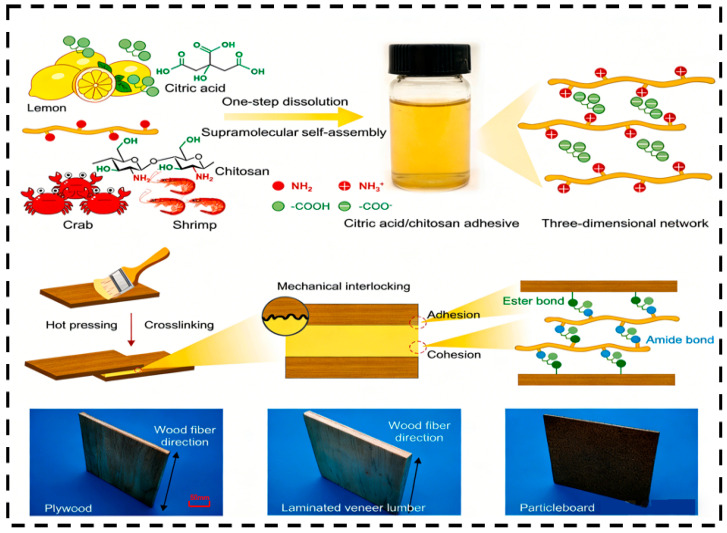
Adhesive Mechanism of Pure Chitosan/Chitin Adhesive [9].

Pure chitin/chitosan adhesives have become a fundamental research direction in the field of biomass adhesives, owing to their advantages such as single-component raw materials, a simple preparation process, and natural biocompatibility. However, their performance is highly dependent on the interfacial interaction mechanisms between molecular chains and the modulation of preparation parameters. Figure 4 illustrates the adhesive mechanism of pure chitin/chitosan adhesives.

From the perspective of the direct gelation mechanism, the adhesive strength of pure chitin/chitosan adhesives primarily originates from hydrogen bonding between molecular chains and substrate surfaces, as well as the unique electrostatic interactions of chitosan. Together, these determine the interfacial bonding strength and the cohesion of the adhesive layer. Hydrogen bonding serves as the core driving force. The abundant hydroxyl (-OH), amino (-NH_2_, in chitosan), and acetamido (-NHCOCH_3_, in chitin) groups on the molecular chains of chitin/chitosan can form strong hydrogen bonds with hydroxyl groups on the surfaces of substrates such as wood and paper. Particularly when the degree of deacetylation (DD) of chitosan increases, the number of amino groups rises, significantly enhancing the hydrogen bond density with substrate hydroxyl groups. For example, the hydrogen bonding energy between chitosan with DD = 80% and pine wood surfaces is 40% higher than that of chitosan with DD = 50%, directly reflected by an increase in dry-state tensile shear strength from 0.8 MPa to 1.2 MPa [97,98].

Electrostatic interaction represents a unique interfacial mechanism for chitosan. Under acidic conditions (pH = 3–5), the amino groups of chitosan become protonated, forming positively charged -NH_3_^+^ ions, which can electrostatically attract negatively charged substrate surfaces (e.g., wood fibers carrying residual pectin). This further enhances interfacial bonding. However, when pH > 6, the amino groups deprotonate, weakening electrostatic interactions and reducing adhesive strength by approximately 30% [9,92].

Additionally, the self-aggregation structure formed via hydrogen bonds between chitin/chitosan molecular chains (e.g., partially crystalline regions in chitosan) can provide a certain degree of cohesion within the adhesive layer. However, due to its high crystallinity (70–80%) and poor solubility, chitin struggles to form a continuous and homogeneous adhesive layer, resulting in lower cohesion compared to chitosan (crystallinity 40–60%) [99,100].

Preparation Process and Parameter Optimization are crucial for achieving the desired performance of pure chintin/chitosan adhesives. This requires establishing a process window, defined by the three core parameters of concentration, pH, and temperature, tailored to different application scenarios.

Concentration directly determines adhesive layer thickness and viscosity. When the chitosan concentration is too low (<2%), the adhesive tends to penetrate excessively into substrate pores, leading to insufficient adhesive on the surface and resulting in a dry-state shear strength of <0.6 MPa. Conversely, an excessively high concentration (>8%) causes a sharp increase in viscosity (>15,000 mPa·s), making coating difficult and the adhesive layer prone to cracking. Practical studies indicate that a chitosan concentration of 3–5% (dissolved in 1% acetic acid) strikes a balance between coatability and bond strength, yielding a viscosity of 2000–6000 mPa·s suitable for the roller-coating process in wood plywood production, with a dry internal bond (IB) strength reaching 0.4–0.5 MPa [20,101].

PH value adjustment must balance electrostatic interactions with molecular chain stability. While acidic conditions (pH = 3–5) promote the protonation of amino groups, enhancing electrostatic attraction, a pH below 3 accelerates the degradation of chitosan chains (e.g., reducing molecular weight from 5 × 10^5^ Da to 2 × 10^5^ Da), thereby weakening cohesion. On the other hand, at pH > 6, chitosan tends to precipitate, leading to an inhomogeneous adhesive layer. Therefore, industrially, chitosan is typically dissolved in acetic acid solution at pH = 4–5, which retains over 80% of protonated amino groups while avoiding chain degradation [9].

Temperature affects both gelation efficiency and adhesive layer curing. During the preparation (dissolution) stage, a temperature of 50–60 °C is optimal, as it shortens the chitosan dissolution time (from 4 h at room temperature to 1.5 h) and prevents chain scission caused by higher temperatures (>80 °C). During the curing (hot-pressing) stage, the temperature must align with wood processing requirements. Hot-pressing at 120–140 °C for 30–60 min promotes hydrogen bond reorganization and moisture evaporation, increasing the dry strength of the adhesive layer by approximately 20%. However, temperatures exceeding 160 °C can induce thermal degradation of chitosan; TGA analysis shows a weight loss of about 10% under such conditions, ultimately leading to a reduction in bond strength [41,71].

From the perspective of fundamental bonding performance and application limitations, pure chitin/chitosan adhesives exhibit a characteristic profile of “adequate performance under dry conditions but weak performance under wet conditions.” Their application is generally confined to scenarios with low-strength and short-service-life requirements.

Regarding dry-state performance, chitosan adhesives outperform chitin. Plywood bonded with a 3% chitosan solution (DD = 75%) can achieve a dry tensile shear strength of 1.0–1.5 MPa and an internal bond (IB) strength of 0.4–0.6 MPa, meeting the requirements for indoor non-load-bearing panels such as decorative particleboard [24]. In contrast, chitin exhibits poorer solubility (often requiring special solvents like N,N-dimethylacetamide/lithium chloride) and tends to form adhesive layers with particulate impurities. Consequently, its dry strength is only 60–70% that of chitosan, limiting its practical applications [49].

Poor wet-state performance is the most critical limitation of the pure system. Its dry-state tensile and shear strength range from 1.0 to 1.5 MPa. Its wet-state retention rate is within 20–30%. It is suitable only for low-strength and short-life scenarios. The abundant polar groups on the molecular chains readily form hydrogen bonds with water molecules, leading to adhesive layer swelling. After 24 h of water immersion, the wet strength retention rate of chitosan adhesives drops to only 20–30%, with IB strength decreasing from approximately 0.5 MPa to 0.1–0.15 MPa [16].

Additionally, the pure system suffers from insufficient bonding strength and poor aging resistance. Compared to urea–formaldehyde resins (dry IB > 1.0 MPa, wet IB > 0.7 MPa), pure chitosan adhesives show a significant strength gap, making them unsuitable for structural wood applications. Furthermore, the amino groups in the molecular chains are prone to oxidation, accelerating aging. Dry strength has been reported to decrease by 15–20% after indoor storage for six months [1,78].

### 4.2. Composite-Modified Adhesive

**Figure 5:**
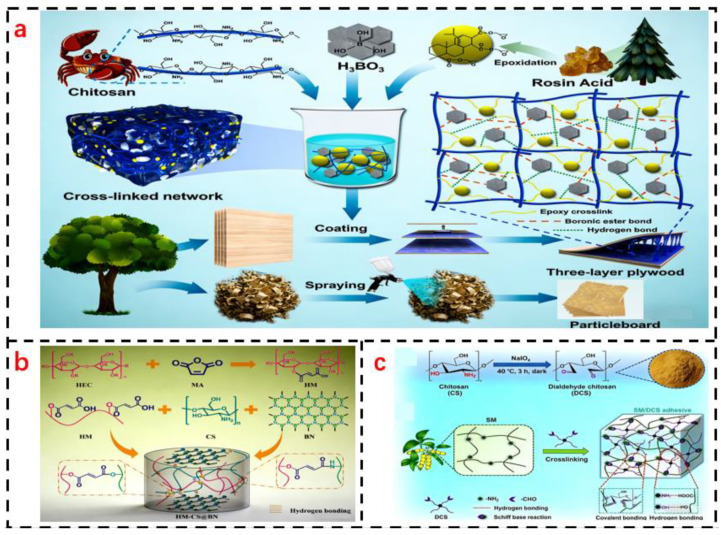
Mechanisms of Action of Composite-Modified Adhesives. (**a**). Preparation process of the CS-BA-ERA adhesive and the manufacturing processes of plywood and particleboard [102]. (**b**). Synthesis mechanism of the HM-CS@BN adhesive [103]. (**c**). Synthesis process of DCS and the reaction mechanism of the SM/DCS adhesive [13].

To overcome the inherent limitations of pure chitin/chitosan adhesives, such as poor water resistance and insufficient bonding strength, composite modification has emerged as a core strategy. By constructing synergistic networks with natural polymers, synthetic polymers, or nanofillers, it is possible to simultaneously optimize interfacial adhesion, cohesive strength of the adhesive layer, and environmental stability. Furthermore, different composite systems are tailored for diverse application scenarios. The synthesis mechanisms and adhesive principles of several composite-modified adhesives are illustrated in Figure 5.

#### 4.2.1. Composite with Natural Polymers

The composite of chitin/chitosan with natural polymers (such as starch, soy protein, lignin, and tannin) offers the primary advantage of utilizing entirely biomass-derived raw materials, ensuring outstanding environmental sustainability. Furthermore, the molecular chains readily form multiple interfacial interactions, including hydrogen bonding and hydrophobic effects, enabling synergistic performance enhancement.

The comprehensive utilization of starch leverages the numerous hydroxyl groups on its molecular chains to form a dense hydrogen bond network with the hydroxyl and amino groups of chitin/chitosan, while its plasticity helps mitigate the brittleness of the adhesive layer. For instance, research by Boussetta et al. [1] demonstrated that combining 3% chitosan with a deacetylation degree of 75% and 10% corn starch, along with 2% citric acid as a cross-linking agent, followed by hot-pressing at 120 °C for 30 min, increased the dry internal bond strength of plywood from 0.5 MPa for pure chitosan to 0.7 MPa. The 24 h wet strength retention rate also improved from 25% to 45%. The incorporation of starch not only enhances hydrogen bonding interactions but also impedes water molecule penetration through its amorphous regions. However, the inherent hydrophilicity of starch tends to increase the moisture absorption of the adhesive layer; this system operates at a dry-state pressure of 0.7 MPa, with a retention rate of 45% in the wet state. The cost is 30% lower than that of pure chitosan, necessitating further optimization through cross-linking or hydrophobic modification [1,9].

Soy protein composite leverages the ionic bonds and hydrogen bonds formed between the amino and carboxyl groups of soy protein and chitosan, while its globular structure enhances the cohesive strength within the adhesive layer. Research by Chen et al. [13] demonstrated that a composite of 5% dialdehyde chitosan and 15% soybean meal resulted in a dry tensile shear strength of 1.8 MPa for the adhesive, with a wet strength retention rate exceeding 50%, attributed to the Schiff base crosslinks formed between the aldehyde groups of the former and the protein amino groups of the latter. Additionally, the inherent antibacterial properties of chitosan reduced wood mold incidence from 80% to 15%. However, soy protein is prone to denaturation, and the composite process requires controlling the temperature below 80 °C to prevent protein aggregation, which can lead to adhesive layer heterogeneity [21,70].

Lignin/tannin composite utilizes the hydrogen bonds formed between the phenolic hydroxyl groups of lignin or tannin and the amino groups of chitosan, while their aromatic ring structures enhance the hydrophobicity and thermal stability of the adhesive layer. Research by Kim et al. [104] indicated that after compounding 3% chitosan with 8% lignosulfonate, the ionic bonds formed between the sulfonate groups of lignin and the amino groups of chitosan reduced the 24 h water absorption rate of the adhesive from 35% (pure chitosan) to 22% and increased the initial thermal decomposition temperature from 250 °C to 275 °C, making it more suitable for high-temperature hot-pressing processes. The incorporation of tannin can further improve the adhesive layer’s resistance to UV aging; the strength reduction rate after six months of indoor storage decreased from 20% to 8% [70,105].

#### 4.2.2. Composite with Synthetic Polymer

Composite with Synthetic Polymers (Polyvinyl Acetate, Epoxy Resin, Isocyanate, etc.): The core principle of this approach lies in leveraging the high strength and hydrophobicity of synthetic polymers. A cross-linked network is constructed through chemical grafting or physical blending to address the performance limitations inherent in purely biomass-based systems.

Composite with Polyvinyl Acctate (PVAc): The PVAc composite is based on the formation of hydrogen bonds between the ester groups of PVAc and the hydroxyl groups of chitosan, while its linear structure concurrently enhances the flexibility of the adhesive layer. Research by Todorović et al. [20] demonstrated that after introducing PVAc segments onto the chitosan molecular chain via free radical grafting (achieving a grafting ratio of 65%), the viscosity of the composite adhesive could be controlled within the range of 5000–8000 mPa·s, making it suitable for roller coating processes. The resulting adhesive exhibited a dry internal bond strength of 0.8 MPa with a wet strength retention rate of 55%. The hydrophobic segments of PVAc effectively impede water molecule penetration, and the grafted structure prevents potential phase separation issues associated with simple blending. However, the incorporation of PVAc reduces the biodegradability of the system; therefore, its addition should be controlled below 15% [74,106].

Composite with Epoxy Resin: The epoxy resin composite leverages the ring-opening reaction between the epoxy groups of the resin and the amino groups of chitosan to form a covalent cross-linked network, thereby significantly enhancing the cohesive strength and water resistance of the adhesive layer. Research by Liu et al. [32] demonstrated that a composite adhesive prepared with 2% chitosan and 5% epoxy resin (E-51), using 1% triethylenetetramine as a curing agent and cured at 140 °C for 60 min, achieved a tensile shear strength of 2.5 MPa. After 24 h of boiling water immersion, the strength retention rate exceeded 70%. This performance is attributed to the covalent cross-linked network’s ability to resist degradation by water molecules, while the rigid structure of the epoxy resin enhances the adhesive layer’s resistance to deformation. However, the inherent brittleness of epoxy resin can lead to a reduction in the toughness of the adhesive layer, typically necessitating the use of toughening agents such as polyetheramines [10,75].

Composite with Isocyanates: The isocyanate composite involves the reaction between the -NCO groups of isocyanates (e.g., MDI) and the hydroxyl and amino groups of chitosan, forming urea and urethane linkages, thereby constructing a high-strength cross-linked network. For instance, compounding 3% chitosan with 4% MDI can increase the crosslink density of the adhesive layer from 2.1 × 10^3^ mol/m^3^ (pure chitosan) to 5.8 × 10^3^ mol/m^3^. This composite achieves a dry internal bond strength of 1.2 MPa and a wet strength retention rate of 65%, meeting the requirements for indoor load-bearing panels [4]. However, due to the significant toxicity of isocyanates, it is necessary to minimize their volatilization by controlling reaction conditions, such as employing low-temperature prepolymerization [4,37].

#### 4.2.3. Nano-Composite Modification

Nanofillers (such as chitin nanocrystals, graphene, and nanocellulose) can form a “nano-reinforcement network” with chitin/chitosan due to their high specific surface area and strong interfacial interactions. This enables significant improvements in mechanical properties and water resistance even at low loadings (typically <5%), while even imparting functional characteristics such as antibacterial or flame-retardant properties.

Chitin and Nanocrystal Composites (CNCs): CNCs are rod-shaped nanoparticles (diameter: 5–20 nm, length: 100–500 nm) prepared via acid hydrolysis of chitin. Their good compatibility with chitosan molecular chains enables the formation of a “rigid reinforcing network” through hydrogen bonding. In the study by Dang et al. [11], the composite of 2% CNCs with 3% chitosan resulted in an increase in the adhesive layer’s elastic modulus from 800 MPa to 1200 MPa, achieving a dry internal bond (IB) strength of 0.9 MPa. Moreover, the crystalline structure of CNCs can hinder water molecule penetration, reducing the 24 h water absorption rate from 30% to 20%. Additionally, the rod-like structure of CNCs enhances the crack resistance of the adhesive layer, mitigating the brittleness issue commonly associated with cured pure chitosan adhesives [11,32].

Chitin and Graphene Composites: The two-dimensional sheet-like structure of graphene can form a “physical barrier layer” within the adhesive, while its high electrical conductivity can impart functional properties to the adhesive. In the study by Liu et al. [32], a composite of 0.5% graphene (reduced graphene oxide) with 3% chitosan was prepared. The sheet-like structure of graphene retards water molecule diffusion, increasing the wet-state internal bond (IB) strength retention rate from 25% to 50%. Furthermore, the π-π interactions between graphene and chitosan enhance the cohesive strength of the adhesive layer, resulting in a dry tensile shear strength of 2.0 MPa. However, graphene tends to agglomerate, necessitating the use of methods such as ultrasonic dispersion (300 W, 30 min) or surface modification (e.g., silane treatment) to improve its dispersibility [32,46].

Chitin and Nanocellulose (NFC) Composites: The hydroxyl groups of NFC can form hydrogen bonds with chitosan, and its fibrous structure enhances the toughness and interfacial bonding strength of the adhesive layer. In the study by Cai et al. [9], a composite of 1% NFC with a 3% chitosan-citric acid system was prepared. The incorporation of NFC increased the elongation at break of the adhesive layer from 5% to 12%, achieving a dry internal bond (IB) strength of 0.8 MPa. Furthermore, the porous structure of NFC can adsorb some water molecules, reducing the 24 h water absorption rate from 18% to 12%. The synergistic effect between NFC and chitosan makes this composite particularly suitable for bonding flexible substrates, such as paper-based packaging materials [3,9].

The performance advantages of different composite systems dictate their distinct suitability for varied application scenarios. Natural polymer composite systems (e.g., chitosan–starch, chitosan–soy protein), due to their fully biomass-derived nature, are suitable for indoor, low-pollution applications such as children’s furniture and decorative panels. However, their wet-state performance remains limited, often requiring the addition of cross-linkers like citric acid or DAC. Synthetic polymer composite systems (e.g., chitosan–PVAc, chitosan–epoxy resin) offer high strength and superior water resistance, making them applicable for load-bearing indoor panels and even certain outdoor scenarios. Nonetheless, a balance between environmental sustainability and performance must be struck, for instance, by controlling the synthetic polymer content to below 20%. Nanocomposite systems (e.g., chitosan–CNCs, chitosan–graphene) achieve significant performance enhancement even at low additive loadings and readily impart functional properties (e.g., reinforcement with CNCs, flame retardancy with graphene). They are thus well-suited for high-value-added applications, including medical dressings and premium packaging.

Three composite modification systems (natural polymer composites, synthetic polymer composites, and nanocomposites) exhibit complementary performance enhancement directions and application scenarios [79]. Natural polymer composite systems (e.g., chitosan–starch, chitosan–soy protein) offer the core advantage of being fully biomass-derived, providing optimal environmental sustainability. Their cost is only 60–70% of that of synthetic polymer composites. However, their wet strength retention generally falls within 45–55%, making them unsuitable for load-bearing applications and more appropriate for indoor low-pollution decorative panels [21].

Synthetic polymer composite systems (e.g., chitosan–PVAc, chitosan–epoxy resin) significantly enhance water resistance through covalent cross-linked networks. For instance, the epoxy resin composite system can achieve a wet strength retention of over 70% after boiling water immersion, with a maximum dry-state tensile shear strength of 2.5 MPa, approaching the performance level of urea–formaldehyde resins [23]. However, the incorporation of synthetic polymers reduces material biodegradability (degradation rate decreases by 30–40%), and certain components (e.g., isocyanates) pose toxicity risks, requiring strict dosage control (typically <20%) [26].

Nanocomposite systems enable efficient reinforcement with low additive amounts (<5%) [107]. For example, chitin nanocrystal (CNC)-based composites can improve the elastic modulus by up to 50%, while graphene-based composites enhance wet strength retention from 25% to 50%, additionally imparting functionalities such as antibacterial and flame-retardant properties [108]. Nevertheless, nano-fillers are prone to agglomeration, necessitating additional processing costs (e.g., ultrasonic dispersion). Currently, such systems are more suitable for high-value applications such as high-end packaging and medical dressings [73].

The three systems emphasize different aspects of performance balance: natural polymer composites prioritize “environmental friendliness–cost” balance, synthetic polymer composites focus on “strength–water resistance” balance, and nanocomposites aim for “multifunctionality–low loading” balance. The selection of an appropriate system should be based on specific application requirements [109].

### 4.3. Chemically Modified Adhesive

**Figure 6:**
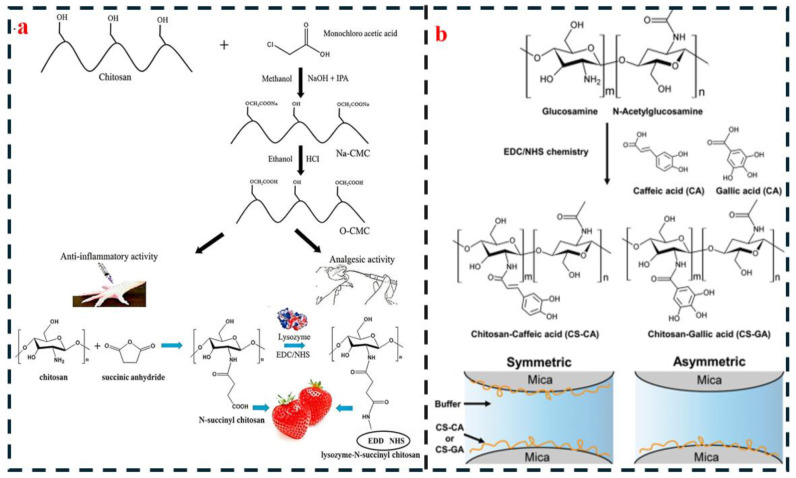
(**a**). Reaction scheme for the carboxylation modification of chitosan [24]. (**b**). Chemical conjugation of caffeic acid (CA) and gallic acid (GA) with chitosan [5].

Chemical modification, by precisely tailoring the molecular architecture of chitin/chitosan to introduce new functional groups or construct cross-linked networks, fundamentally overcomes the performance limitations of pure or simple composite systems. This approach represents a crucial strategy for advancing these materials from “basic adhesion” to “high-performance, multifunctional” applications. The primary modification methods and their characteristics are summarized in Figure 6.

#### 4.3.1. Crosslinking Modification

Cross-linking modification involves covalent reactions between cross-linking agents and the hydroxyl or amino groups on chitin/chitosan molecular chains, forming a three-dimensional cross-linked network. This network reduces the space available for molecular chain swelling while simultaneously enhancing the cohesive strength of the adhesive layer, making it a key strategy for addressing poor water resistance. Different cross-linking agents are suitable for distinct application scenarios due to variations in their reactivity and toxicity.

Genipin cross-linking utilizes genipin, a natural cross-linking agent derived from gardenia fruit. Its iridoid structure undergoes nucleophilic addition reactions with the amino groups of chitosan, forming stable imine and amide bonds. This reaction is non-toxic, making it particularly suitable for biomedical and food-contact applications. Research by Silvestre et al. [16] demonstrates that after the reaction of 2% genipin with 3% chitosan (85% deacetylation degree), the cross-linking density increases from 1.8 × 10^3^ mol/m^3^ for pure chitosan to 4.5 × 10^3^ mol/m^3^. Consequently, the dry tensile shear strength of the adhesive on wood improves from 1.2 MPa to 1.8 MPa, and its 24 h wet strength retention rate increases from 25% to 60%. The genipin-crosslinked adhesive layer exhibits a survival rate of 90% for L929 cells, meeting the adhesion requirements for medical dressings [14]. However, the relatively slow reaction rate of genipin (typically requiring 4 h at 40 °C) and its higher cost limit its large-scale application.

Citric acid, employed as a cost-effective and eco-friendly cross-linking agent, enables dual “covalent-ionic” cross-linking. Its carboxyl groups can undergo esterification reactions with the hydroxyl groups of chitosan while also forming ionic bonds with amino groups. When the hydroxyl (-OH) and amino (-NH_2_) groups of chitosan undergo esterification reactions with oxy-acids (such as phosphoric acid, sulfuric acid, etc.) or acid derivatives (such as citric acid, glutaraldehyde, etc.), new functional groups (e.g., phosphate ester, sulfate ester, citrate ester groups, etc.) are introduced into the molecular chains. These functional groups not only increase the polarity and charge density of the molecules, optimizing electrostatic interactions and complexation ability with target adsorbates (such as heavy metal ions, dye molecules, organic pollutants, etc.) but also enhance the accessibility and capacity of adsorption sites by altering the spatial conformation of chitosan, increasing the specific surface area, or constructing cross-linked networks. Meanwhile, esterification modification can adjust the hydrophilicity/hydrophobicity of chitosan, improve its stability in different media, and reduce swelling or dissolution losses during adsorption, thereby comprehensively improving its adsorption selectivity, adsorption kinetics, and regeneration performance [9,21]. This mechanism makes it suitable for low-cost applications such as wood processing. Research by Cai et al. [9] demonstrates that a composite of 5% citric acid with 4% chitosan, when thermally cured at 120 °C, forms ester bonds between the carboxyl groups of citric acid and the hydroxyl groups of chitosan. This reduces the 24 h water absorption of the adhesive layer from 35% to 18%, achieving a dry internal bond strength of 0.7 MPa and a wet strength retention rate of 50%. Furthermore, citric acid can adjust the pH of the adhesive solution to 4–5, promoting chitosan dissolution and thereby eliminating the need for additional acid additives [9,16].

Epichlorohydrin cross-linking involves the ring-opening reaction of its epoxy groups with the amino and hydroxyl groups of chitosan, forming ether bonds and additional hydroxyl groups to construct a high cross-linking density network. This method provides outstanding water resistance; however, care must be taken due to the inherent toxicity of epichlorohydrin, necessitating strict control of its residual amount. In the study by Liu et al. [32], after the reaction of 3% epichlorohydrin with 3% chitosan, thermogravimetric analysis (TGA) of the adhesive layer showed a reduction in weight loss at 200 °C from 15% to 5%. The adhesive retained 75% of its wet internal bond strength after 2 h of boiling water immersion, meeting the bonding requirements for some outdoor wood applications. Nonetheless, during preparation, ethanol washing (three times) is essential to ensure the residual epichlorohydrin content remains below 0.1% [10,32].

#### 4.3.2. Graft Copolymer Modification

Graft copolymerization involves attaching synthetic polymer chains (such as polyvinyl acetate or acrylic-based polymers) onto the backbone of chitin/chitosan via methods like free-radical polymerization. This approach leverages the flexibility and hydrophobicity of the grafted chains to tailor the mechanical properties and water resistance of the adhesive layer, while simultaneously avoiding the phase separation issues commonly associated with simple blending. The reagents used in graft polymerization and their functions are shown in Table 5.

Grafting polyvinyl acetate (PVAc) is a common method to improve the properties of chitosan-based adhesives by leveraging the good flexibility and adhesion of PVAc segments. This reaction typically employs ammonium persulfate (APS) as the initiator, which facilitates the graft copolymerization onto the hydroxyl groups adjacent to the amino groups of chitosan, thereby enhancing the flexibility of the adhesive layer and its compatibility with substrates. Experiments by Todorovic et al. [20] show that when using 0.5% APS and reacting chitosan with vinyl acetate at a mass ratio of 1:3 at 60 °C for 4 h, a grafting ratio of up to 65% can be achieved. The viscosity of the modified adhesive increases from 3000 mPa·s for pure chitosan to 6500 mPa·s, making it more suitable for the roller-coating process used in plywood production. Its dry internal bond strength reaches 0.8 MPa, with a wet strength retention rate of 55%. Furthermore, the introduction of PVAc segments increases the elongation at break of the adhesive layer from 5% to 15%, effectively preventing cracking after curing [20,106].

Grafting acrylic monomers, such as acrylic acid (AA) or methyl methacrylate (MMA), introduces carboxyl or ester groups into the chitosan molecular structure. This not only enhances polar interactions between the adhesive layer and substrates but also improves the material’s hydrophobicity. Research by Chen et al. [24] demonstrates that using an AIBN initiator (0.3%), with chitosan and AA in a mass ratio of 1:2 reacting at 70 °C for 3 h, a grafting ratio of up to 50% can be achieved. The carboxyl groups on the grafted chains form hydrogen bonds with hydroxyl groups on wood, resulting in an adhesive with a high dry tensile shear strength of 2.0 MPa. Concurrently, the hydrophobic effect of the AA-grafted segments reduces the 24 h water absorption rate from 30% to 22%. When MMA is used for grafting, the adhesive exhibits even better hydrophobicity (water absorption can decrease to 18%), though with a slight reduction in flexibility [24,37].

#### 4.3.3. Functional Modification

Functional modification introduces specific functional groups (such as catechol, phosphate groups, or maleic anhydride) into chitin/chitosan adhesives. This endows the adhesives with both bonding performance and additional functionalities (e.g., wet adhesion, flame retardancy, antibacterial properties), making them suitable for high-end or specialized application requirements.

Catechol grafting is a modification strategy inspired by the adhesion mechanism of mussel byssus. The catechol (ortho-dihydroxybenzene) groups can form strong interfacial interactions through oxidative cross-linking, leading to particularly excellent performance in wet environments. As demonstrated in the study by Lee et al. [5], after grafting dopamine onto chitosan molecular chains (with a grafting ratio of 20%) via carbodiimide activation, the resulting adhesive achieved a wet tensile shear strength of 1.2 MPa on wood underwater (pH = 7), which is five times that of pure chitosan. Furthermore, the oxidative cross-linking of catechol groups can form a dense film on the adhesive surface, effectively impeding water penetration [5,110].

Phosphorylation modification typically involves reacting chitosan hydroxyl groups with reagents such as phosphoric acid or sodium dihydrogen phosphate to introduce phosphate groups. This process not only imparts flame-retardant properties to the adhesive but also enhances interfacial bonding through the coordination of phosphate groups with metal ions. For instance, after reacting chitosan with phosphoric acid at a molar ratio of 1:2 at 80 °C for 3 h, the degree of phosphorylation can reach 30%. The modified adhesive exhibits an increase in the Limiting Oxygen Index (LOI) from 21% (pure chitosan) to 28%, meeting the UL94 V-1 flame-retardant rating [12]. Simultaneously, the phosphate groups can form coordination bonds with Ca^2+^ ions present in wood, resulting in a dry internal bond strength of 0.9 MPa. This makes the adhesive suitable for the production of flame-retardant wood composite panels [106].

Maleation modification utilizes the ring-opening reaction between maleic anhydride and the hydroxyl groups of chitosan to introduce carboxyl groups and double bonds. The introduced carboxyl groups enhance polar interactions, while the double bonds offer the possibility for subsequent cross-linking to further improve performance. Research by Zeng et al. [72] shows that under conditions of a maleic anhydride to chitosan mass ratio of 1:4 and reaction at 100 °C for 2 h, a maleation degree of up to 40% can be achieved. When this modified product is combined with glucose and cured at 140 °C, free-radical cross-linking of the double bonds occurs. The resulting adhesive layer retains 80% of its wet internal bond strength after 2 h of boiling water immersion, with a dry strength of 1.1 MPa. This successfully overcomes the application limitation of pure systems being intolerant to boiling water [71,72].

### 4.4. Biomineralization and Biomimetic Adhesive Design

Biomineralization and bioinspired design represent cutting-edge directions for advancing chitin/chitosan-based adhesives toward “high-performance and multifunctional” applications. The former involves constructing an “organic–inorganic” composite reinforcement network by mimicking the synergistic interaction between inorganic phases (e.g., hydroxyapatite, calcium carbonate) and organic matrices within biological systems. The latter draws inspiration from the adhesion mechanisms of natural organisms (such as mussels and lobsters) to optimize interfacial interaction patterns, thereby overcoming the performance limitations of conventional adhesives in wet or complex environments.

#### 4.4.1. Simulated Biological Adhesion Mechanism

**Figure 7:**
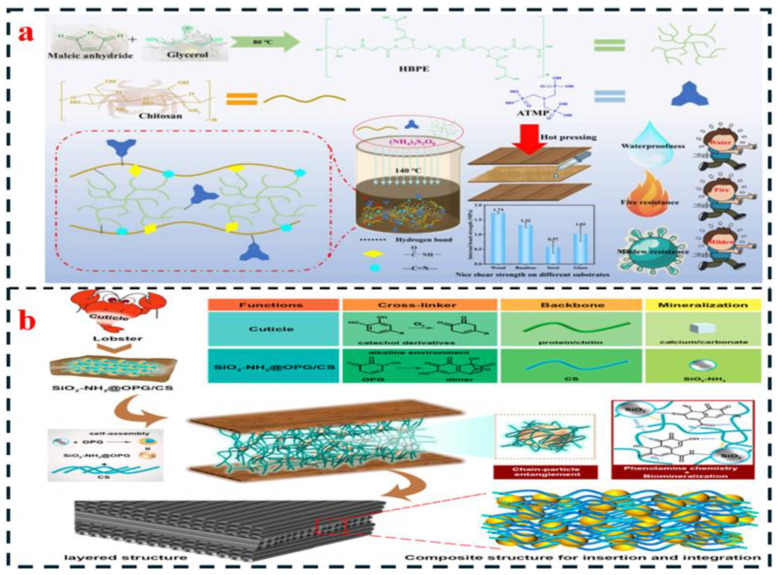
(**a**). Schematic diagram of the fabrication process for the HBPE-CS adhesive and its water-resistant, flame-retardant, and anti-mold properties [111]. (**b**). Bioinspired design and chemical mechanism of the siloxane-carboxyl-OPG/polyacrylate adhesive, mimicking the exoskeleton of marine arthropods [46].

The efficient adhesion mechanisms observed in natural organisms provide natural paradigms for the design of chitin/chitosan-based adhesives. The core approach involves molecular structure biomimicry to replicate the synergistic effect of “chemical interactions—physical structures” inherent to biological adhesion, which is particularly prominent in wet and dynamic environments, as illustrated in Figure 7.

Mussel byssus achieves strong interfacial adhesion with substrates through the oxidative crosslinking of catechol groups, a mechanism widely adopted to enhance the wet adhesion performance of chitin/chitosan. In the study by Lee et al. [5], dopamine (containing catechol groups) was grafted onto chitosan molecular chains (with a grafting ratio of 20–30%) via a carbodiimide activation method. The resulting catechol-grafted chitosan adhesive exhibited a wet tensile shear strength of 1.2–1.5 MPa on wood under aqueous conditions (pH = 7), which is 5–6 times higher than that of pure chitosan. The catechol groups can be oxidized in moist environments to form quinone structures, which subsequently undergo nucleophilic addition reactions with hydroxyl and amino groups on the wood surface. Concurrently, oxidative crosslinking between catechol groups within the molecular chains forms a dense adhesive layer that impedes water penetration. Further studies indicate that grafting with pyrogallol (1,2,3-trihydroxybenzene), which mimics stronger biological adhesion, can increase the wet strength by an additional 20%. However, its higher cost makes it more suitable for high-end biomedical applications, such as wound closure adhesives [110].

The “layered fiber-mineral” structure of the lobster exoskeleton exhibits exceptional mechanical strength, and its adhesion mechanism inspires the structural biomimicry of chitin/chitosan adhesives. In the study by Ni et al. [46], the “chitin fiber–calcium carbonate” composite structure of the lobster exoskeleton was mimicked. Chitin nanocrystals (CNCs, simulating lobster fibers) and calcium carbonate nanoparticles (simulating the mineral phase) were incorporated into a chitosan matrix to construct a dual-reinforcement network of “nanofibers–inorganic particles”. The results demonstrated that the composite adhesive containing 3% CNCs and 2% calcium carbonate achieved a dry tensile shear strength of 2.5 MPa. Its elastic modulus increased to 1500 MPa, which is 2.3 times that of pure chitosan. Furthermore, the calcium carbonate particles can form coordination bonds with the amino groups of chitosan, reducing molecular chain swelling. This composite retained 70% of its strength after 24 h in a wet state, making it suitable for load-bearing wood bonding applications [37,46].

Beyond mussels and lobsters, other bioinspired approaches have drawn inspiration from the “microstructural adhesion” mechanism of insect tarsi. By employing a templating method, micro/nano-protrusion structures are constructed on the surface of chitosan adhesive layers to increase the contact area with substrates. In the study by Sajomsang [47], using the surface microstructure of cicada slough as a template, a chitosan adhesive with micrometer-scale protrusions on its surface was fabricated. This adhesive achieved a dry internal bond (IB) strength of 0.9 MPa on rough wood surfaces, representing a 30% improvement compared to a smooth adhesive layer. This synergistic model of “structural biomimicry + chemical adhesion” provides a new strategy for bonding complex substrates.

#### 4.4.2. Strategies for Enhancing Biomineralization

**Figure 8:**
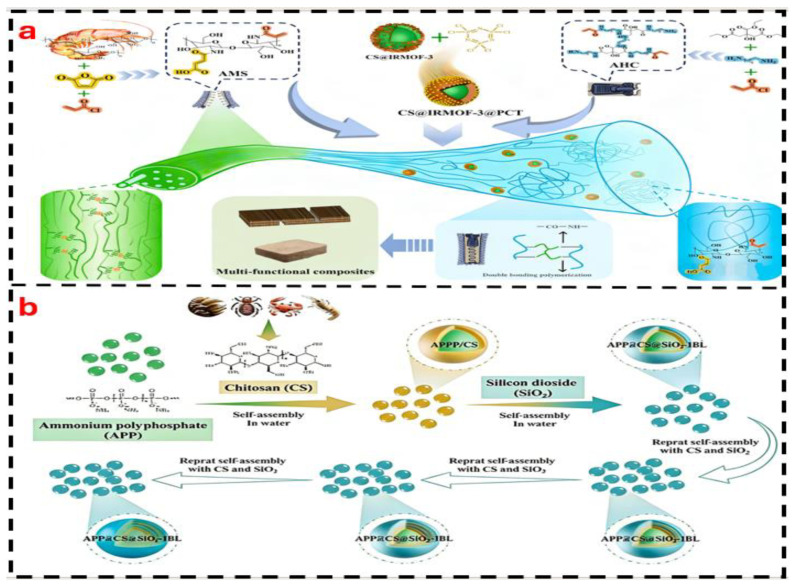
(**a**,**b**) Preparation Process and Mechanism of Biomineralization [32].

Biomineralization enhances the mechanical properties, water resistance, and functional characteristics (e.g., biocompatibility, flame retardancy) of adhesives by incorporating an inorganic phase (such as hydroxyapatite, calcium phosphate, or silica) into a chitin/chitosan organic matrix. This process leverages the synergistic effect between the high strength of the inorganic phase and the flexibility of the organic phase. The preparation techniques and mechanisms of biomineralization are illustrated in Figure 8. 

Hydroxyapatite (HA) mineralization holds significant potential in medical adhesive applications, benefiting from HA’s role as the primary inorganic component of human bone, which aligns with the inherent biocompatibility of chitin/chitosan. According to research by Zhang et al. [14], HA nanoparticles with diameters of 50–100 nm were generated in a chitosan solution via in situ mineralization. When the mass ratio of HA to chitosan was 1:4, the composite adhesive achieved a shear strength of 1.8 MPa on titanium alloy dental implants. Concurrently, the presence of HA was found to promote osteoblast adhesion, resulting in a cell viability rate of 92%, thereby fully meeting the requirements for dental restoration. The synergistic enhancement mechanism primarily stems from the hydrogen bonding interactions between the hydroxyl groups on the HA surface and the amino groups of chitosan. Furthermore, the rigid structure of HA effectively enhances the cohesive strength of the adhesive layer and decelerates the material degradation rate [14,23].

Calcium carbonate (CaCO_3_) mineralization, as a low-cost and facile preparation method, is suitable for general adhesive applications such as in wood and paper bonding. For instance, using a gas diffusion method (by introducing CO_2_) to generate calcite-type CaCO_3_ nanoparticles in situ within a chitosan solution, the addition of 2% CaCO_3_ increased the dry internal bond strength of the composite adhesive from 0.5 MPa (pure chitosan) to 0.9 MPa, while the 24 h water absorption rate decreased from 35% to 22% [32]. This improvement is attributed to the fact that CaCO_3_ not only physically reinforces the adhesive layer through filler effects but also enables its surface calcium ions to form coordination bonds with the carboxyl groups of maleic acid-modified chitosan, thereby constructing a dual cross-linked network based on “hydrogen bonds and coordination bonds.” When further combined with epichlorohydrin cross-linking, the wet strength retention rate of the material can reach 65% [32,71].

Silica (SiO_2_) mineralization leverages its high specific surface area and excellent weatherability to significantly enhance the thermal stability and outdoor applicability of chitin/chitosan-based adhesives. In one study, a sol–gel method was used to generate SiO_2_ sol with particle sizes of 20–50 nm within a chitosan matrix. When the mass ratio of SiO_2_ to chitosan was 1:5, the initial thermal decomposition temperature (T_5_%) of the composite adhesive increased from 250 °C to 285 °C [10]. Its resistance to UV aging was also significantly improved, with the strength reduction rate decreasing from 20% to 8% after 12 months of indoor storage. Furthermore, the hydrophobic surface of SiO_2_ effectively reduces water molecule adsorption, lowering the 24 h water absorption rate to 18%, making it suitable for certain outdoor wood bonding applications.

Multi-Phase Synergistic Mineralization Strategy: The multi-phase synergistic mineralization strategy aims to achieve complementary functionalities by compounding two or more inorganic phases, thereby overcoming the limited enhancement effect of a single inorganic phase. For example, compounding cellulose nanocrystals (CNCs, an organic reinforcing phase) with hydroxyapatite (HA, an inorganic reinforcing phase) at a mass ratio of 2:1 within a chitosan matrix can form a “fiber–particle” dual-reinforcement network [32]. This composite adhesive achieves a dry tensile shear strength of up to 2.8 MPa with a wet strength retention rate of 75%. By simultaneously offering biocompatibility and high strength, it presents dual potential for applications in orthopedic repair and structural wood adhesives.

Biomineralization and Bioinspired Design of Chitin/Chitosan-Based Adhesives: The fundamental principle of biomineralization and bioinspired design in chitin/chitosan-based adhesives lies in “learning from nature.” Bioinspired design primarily focuses on optimizing chemical interactions through molecular structure design (e.g., oxidative crosslinking of catechol groups), while biomineralization emphasizes the interfacial synergy between the organic matrix and inorganic phases (e.g., hydroxyapatite, calcium carbonate) via hydrogen bonds, coordination bonds, and other interactions. Both approaches aim to enhance the interfacial bonding strength between the adhesive and the substrate, and require customization based on specific application scenarios. For medical applications, systems with inherent biocompatibility, such as HA (hydroxyapatite) and catechol-based biomimetics, are prioritized. For wood bonding, systems emphasizing high strength and low cost, such as CaCO_3_ and lobster-inspired designs, are preferred. For outdoor applications, systems with excellent weather resistance, such as SiO_2_ mineralization, are more suitable. Furthermore, the adoption of green processes like in situ mineralization and the use of natural biomimetic agents aligns with industry development trends.

Future research needs to further break through bottlenecks such as the dispersibility of inorganic phases and the scalable fabrication of bioinspired structures. For instance, modifying chitosan molecular chains (e.g., by introducing carboxyl or phosphate groups) can enhance their ability to disperse inorganic phases. Alternatively, developing processes like roller pressing, mold pressing, and photolithography with thermal reflow can facilitate the industrial-scale production of bioinspired microstructures. Concurrently, integrating nanotechnology, synthetic biology, and artificial intelligence can optimize the mineralization process and structural design, accelerating the translation of these high-performance adhesives from laboratory research to practical applications.

Biomineralization and bioinspired design, as two cutting-edge technologies, exhibit distinct emphases in their performance enhancement pathways and application suitability. Bioinspired design focuses on “interface interaction optimization” by mimicking biological adhesion mechanisms through molecular structure engineering [6]. For instance, catechol-grafted systems, which emulate mussel adhesion, achieve a wet adhesion strength of 1.2–1.5 MPa in underwater conditions (pH = 7)—3–5 times higher than that of conventional modified systems—while maintaining excellent biocompatibility (cell viability > 90%) [32]. However, catechol groups are susceptible to oxidation under ultraviolet light, leading to a strength loss of up to 25% over six months. Therefore, such systems are more suitable for short-term adhesion in humid environments, such as wound dressings. On the other hand, the “fiber–particle” dual-reinforcement network inspired by lobster exoskeleton results in a dry-state tensile shear strength of 2.5 MPa and an increase in elastic modulus of up to 130%, representing the optimal mechanical performance among such systems. Nevertheless, replicating this microstructure is process-intensive and challenging to scale up, limiting its current application to structural wood adhesives [18].

In contrast, biomineralization emphasizes “organic–inorganic synergistic reinforcement”. Hydroxyapatite (HA) mineralized systems exhibit outstanding biocompatibility and osteoconductivity, achieving a shear strength of 1.8 MPa on titanium alloy dental implants, making them an ideal choice for medical adhesives. Calcium carbonate (CaCO_3_) mineralized systems offer the lowest cost (only one-fifth that of HA mineralization) and reduce the 24 h water absorption rate to 22%, rendering them suitable for general-purpose wood and paper bonding [105]. Silica (SiO_2_) mineralized systems demonstrate the best thermal stability (with an initial decomposition temperature increased by 35 °C) and a UV-aging strength loss of only 8%, making them well-suited for outdoor applications [12].

The two technologies face different core challenges: bioinspired design must overcome the bottleneck in scaling up the fabrication of biomimetic structures, while biomineralization needs to address the issue of inorganic phase inhomogeneity (agglomeration rates can reach 30–40%) [8]. A multiphase synergistic strategy—such as combined mineralization with chitin nanocrystals (CNCs) and HA—can integrate their respective advantages, achieving a dry strength of 2.8 MPa and a wet strength retention of 75%. This approach provides a promising new direction for the development of high-performance adhesives [3].

## 5. Conclusions and Future Outlook

### 5.1. Conclusions

Research Progress and Mechanisms of Chitin/Chitosan-Based Biomass Adhesives

The core advancements in the research of chitin/chitosan-based biomass adhesives revolve around “overcoming performance deficiencies” and “expanding application scenarios.” The underlying principle involves the precise regulation of molecular structure and interfacial interactions through diverse modification technologies, enabling an upgrade from “basic adhesion” to “high-performance and multifunctional” materials.

At the level of modification technologies, chemical modification and composite modification form a synergistic system. Crosslinking modification constructs three-dimensional networks using agents such as genipin, citric acid, and epichlorohydrin. For instance, genipin crosslinking increased the wet strength retention rate of chitosan adhesive from 25% to 60%. Citric acid achieves dual “covalent-ionic” crosslinking, reducing the 24 h water absorption rate to 18%. Graft copolymerization introduces chain segments like PVAc and acrylics to balance flexibility and hydrophobicity. PVAc grafting increased the adhesive layer’s elongation at break from 5% to 15%, while acrylic grafting resulted in a dry tensile shear strength of 2.0 MPa. Functionalization modifications impart specialized properties: catechol grafting mimics mussel adhesion, achieving an underwater wet strength of 1.2 MPa, and phosphorylation increases the Limiting Oxygen Index (LOI) to 28%.

Composite modification achieves synergistic enhancement through “organic–organic” and “organic–inorganic” strategies. For example, the composite of chitosan and soy protein, utilizing Schiff base crosslinking, achieves a dry strength of 1.8 MPa with an antibacterial rate exceeding 85%. Composites of chitin nanocrystals (CNCs) and calcium carbonate build a “fiber–particle” network, elevating the dry strength to 2.5 MPa.

The key role of these modification technologies is twofold. On one hand, they enhance the cohesive strength of the adhesive layer through covalent crosslinking and graft chain segment regulation, addressing the issue of “insufficient bond strength.” On the other hand, they optimize interfacial interactions through hydrophobic segments and inorganic phase barriers, overcoming the bottleneck of “poor water resistance.” Simultaneously, they introduce functionalities such as antibacterial and flame-retardant properties, enabling the adhesive to be adapted for diverse scenarios including wood bonding and biomedical applications. Future research should focus on three key priority directions. First, the efficient and cost-effective development of green modification processes is essential. Current green technologies such as enzymatic methods and microwave-assisted processes face challenges like high enzyme costs and strong equipment dependency. There is a need to develop “one-step” extraction–modification processes (e.g., simultaneous chitin enzymatic hydrolysis and chitosan grafting), optimize parameters for supercritical CO_2_-assisted crosslinking, and reduce energy consumption and reagent usage to facilitate the scaling of green technologies from the laboratory to industrial production. Second, the synergistic optimization of comprehensive performance must be addressed. Existing modifications often emphasize a single property; thus, molecular design should aim to balance “strength, water resistance, and stability.” For example, incorporating lignin into catechol-grafted systems can preserve wet adhesion performance while enhancing UV aging resistance, or using nano-composites (e.g., CNCs + SiO_2_) to simultaneously improve mechanical properties and durability. Finally, multifunctional integrated design should be pursued, incorporating intelligent responsive characteristics. This includes developing pH-responsive medical adhesives (adapted to wound healing cycles) and temperature-sensitive wood adhesives (compatible with hot-pressing processes), while integrating functionalities such as antibacterial and flame-retardant properties to meet the demands of high-end applications. Industrialization breakthroughs need to target three key pathways. First, comprehensive resource utilization must be promoted on the raw material front. For example, protein residues remaining after chitin extraction from shrimp and crab shells can be used to prepare cross-linking agents. Combining these with agricultural and forestry wastes such as lignin and tannin for compounding with chitosan can reduce raw material costs and achieve “zero waste of by-products.” Second, the production side must focus on equipment adaptation and process standardization. To address the viscosity sensitivity of chitosan solutions, specialized application equipment such as precision temperature-controlled roller coaters should be developed. Furthermore, establishing continuous production lines spanning from raw material extraction to adhesive preparation is crucial. Concurrently, the industry should drive the formulation of dedicated testing standards, including evaluation methods for biodegradability and antibacterial properties. Finally, on the application end, efforts should concentrate on scenario segmentation and market cultivation. In the wood sector, the focus can be on high-end low-formaldehyde boards, such as those for children’s furniture. In the medical field, breakthroughs can be targeted at niche markets like dental restoration and wound dressings. By adopting a strategy that combines “performance parity with traditional adhesives and a premium for green characteristics,” market trust can be gradually established, ultimately achieving industrial-scale substitution.

### 5.2. Current Challenges

Although significant progress has been made in the performance and application of chitin/chitosan-based biomass adhesives, transitioning from laboratory research to widespread industrial adoption faces considerable challenges. First, significant bottlenecks exist in controlling production costs and scaling up manufacturing technologies. The current extraction of chitin primarily relies on traditional acid–alkali methods, which involve substantial chemical usage (e.g., 10% hydrochloric acid for demineralization and 30% sodium hydroxide for deacetylation). Subsequent wastewater treatment accounts for 30–40% of the total production cost. While greener extraction technologies like enzymatic methods are more environmentally friendly, the cost of enzymes is 2–3 times higher than traditional chemical reagents. Moreover, fermentation-based extraction cycles can take up to 72 h, far exceeding the 6–8 h required for acid–alkali methods, making it difficult to align with the pace of continuous industrial production. Additionally, viscosity control during adhesive preparation poses difficulties, as chitosan solution viscosity is highly sensitive to concentration and pH. During large-scale application, this can lead to issues like “localized gelation” or “excessive penetration,” necessitating specialized equipment modifications and further increasing capital investment for enterprises.

Second, achieving a synergistic optimization of comprehensive properties is highly challenging. Existing modifications often focus on enhancing a single property. For instance, while catechol grafting can increase wet strength to 1.2 MPa, it often reduces the adhesive layer’s resistance to UV aging (the strength loss rate after 6 months increases from 15% to 25%). Phosphorylation modification can achieve a Limiting Oxygen Index (LOI) of 28%, but it typically leads to a 10–15% reduction in dry bond strength compared to pure chitosan. Particularly in wood applications, it is difficult to simultaneously meet industrial requirements such as “dry IB > 0.8 MPa, wet IB retention > 60%, and strength loss after 5 years of aging < 10%.” The overall balance of properties still falls short of traditional adhesives like urea–formaldehyde resin.

Finally, there is a lack of dedicated standard systems and industry specifications. Currently, no specific testing standards exist for chitin/chitosan-based biomass adhesives. For example, bond strength testing often references the national standard for wood adhesives (GB/T 4897-2015), but this fails to account for their unique characteristics like biodegradability and antibacterial properties. The absence of industry standards leads to inconsistent product quality. Some companies claim “fully biodegradable” without specifying degradation cycles or environmental conditions, hindering the establishment of market trust.

### 5.3. Future Development Direction

To address current challenges and align with the trends in sustainable materials, future research on chitin/chitosan-based biomass adhesives should focus on three key directions. Firstly, the development and application of green modification technologies is paramount, with an emphasis on breakthrough solvent-free and low-energy processes. For instance, employing microwave-assisted grafting (300–500 W power) to replace conventional heating can reduce grafting reaction time from 4 h to 1 h while lowering energy consumption by 40%. Exploring supercritical CO_2_-assisted crosslinking leverages its high permeability to achieve uniform dispersion of crosslinkers like genipin or citric acid, eliminating organic solvent residues and enhancing crosslinking efficiency. Furthermore, developing “one-pot” extraction–modification processes, such as synchronizing chitin enzymatic hydrolysis with chitosan grafting, can streamline production steps and reduce energy input.

Secondly, the design of multifunctional integrated adhesives through molecular structure modulation to achieve synergistic “adhesion-functionality” performance is crucial. Examples include incorporating phosphate groups into catechol-grafted chitosan, which can maintain an underwater adhesive strength of 1.2 MPa while conferring flame-retardant properties with a Limiting Oxygen Index (LOI) > 27%, making it suitable for outdoor, humid environments with fire-safety requirements. Designing pH-responsive adhesives that exploit the protonation differences of chitosan amino groups under varying pH conditions enables “rapid adhesion in acidic environments and gradual degradation in neutral environments,” meeting the needs of wound dressings for “instant fixation and progressive healing.” Combining the reinforcing effect of chitin nanocrystals with the inherent antibacterial properties of chitosan allows for the development of integrated “high-strength, antibacterial, biodegradable” wood adhesives, reducing the need for post-treatment preservatives.

Finally, achieving the comprehensive resource utilization of biomass waste by constructing synergistic “chitin/chitosan-other biomass” systems is essential. For example, utilizing protein residues left after chitin extraction from shrimp/crab shells to prepare protein-based crosslinkers for compounding with chitosan can achieve a dry internal bond (IB) strength of 0.9 MPa, realizing “zero waste” of by-products. Compounding pulping waste like lignin and tannin with chitosan leverages the hydrophobicity of lignin to improve water resistance while reducing raw material costs, promoting the high-value utilization of agricultural and forestry waste.

## Figures and Tables

**Table 1:** Primary Sources, Distribution, and Characteristics of Chitin/Chitosan.

Raw Material Category	Specific Source	Main Distribution Acquisition Pathway	Chitin/Chitosan Content (dry weight)	Advantages & Characteristics	References
Marine Biomass Waste	Shrimp shells (e.g., Penaeus, Macrobrachium)	Seafood processing waste; global annual production exceeds 6 million tons, concentrated in coastal fishery processing zones	Chitin: 15–25%; Chitosan (after deacetylation): 8–18%	Centralized source, large output, mature extraction process, high industrial potential	[41,42,43]
	Crab shells (e.g., Portunus, Scylla)	Seafood processing waste; annual production exceeds 3 million tons; chitin content higher than shrimp shells	Chitin: 20–30%; Chitosan (after deacetylation): 12–22%	High chitin purity, lower impurity (protein, mineral) content, relatively low extraction cost	[1,42,44,45]
	Lobster shells (e.g., Homarus americanus)	High-end seafood processing waste; mainly distributed in North America, Europe, and coastal China	Chitin: 18–28%; Chitosan (after deacetylation): 10–20%	Large chitin molecular weight, excellent mechanical properties, suitable for high-performance adhesive raw materials	[43,46]
Other Sources	Cicada slough (e.g., Cryptotympana atrata)	Terrestrial insect waste; widely distributed in temperate and subtropical regions; obtainable via manual collection or farming	Chitin: 10–18%; Chitosan (after deacetylation): 6–14%	Free from marine salt impurities; no complex desalting steps required during extraction; product purity easily controlled	[42,47]
	Fungal cell walls (e.g., yeast, mold)	Microbial fermentation industry waste (e.g., Saccharomyces cerevisiae residue); artificially cultured fungi (e.g., Aspergillus niger)	Chitin: 5–12%; Chitosan (naturally present in some fungi): 3–8%	Scalable production via microbial fermentation; not limited by season or geography; composition easily adjustable	[48,49]
	Squid cartilage, Cuttlebone	Cephalopod seafood processing waste; relatively low yield; concentrated in deep-sea fishery processing zones	Chitin: 8–15%; Chitosan (after deacetylation): 5–10%	Unique chitin structure (low crystallinity), good solubility, easy modification, suitable for biomedical adhesives	[43,49]

**Table 2:** Different Extraction Methods and Their Characteristics.

Process Category	Specific Process Step	Core Parameters	Process Characteristics	Application Suitability (Adhesive Scenarios)	References
Traditional Extraction Methods	Demineralization (Acid/Alkali Method)	Acid type: Hydrochloric acid/Sulfuric acid; Concentration: 5–15%; Temperature: 20–80 °C; Time: 1–6 h; Solid-to-liquid ratio: 1:10–1:20	High efficiency in removing calcium salts and other minerals (removal rate > 95%), low cost, industrially mature, but high acid consumption, prone to equipment corrosion and wastewater pollution	Suitable for large-scale production of general wood adhesive raw materials, e.g., chitin extraction from shrimp/crab shells; subsequent neutralization required to treat residual acid	[52,53]
	Deproteinization (Acid/Alkali Method)	Alkali type: Sodium hydroxide; Concentration: 4–10%; Temperature: 60–100 °C; Time: 2–8 h; Solid-to-liquid ratio: 1:8–1:15	Protein removal rate > 90%, process easily controlled, but high temperature and strong alkali may degrade chitin molecular chains and reduce molecular weight	Suitable for preparing medium- to low-strength adhesives (e.g., paper bonding); subsequent molecular weight adjustment needed to avoid insufficient bonding strength	[54,55]
	Deacetylation (Preparation of Chitosan)	Alkali type: Sodium hydroxide; Concentration: 30–50%; Temperature: 80–120 °C; Time: 2–10 h; Stirring rate: 100–300 rpm	Controllable degree of deacetylation (DD) (50–95%), but energy-intensive, difficult alkali recovery, and may cause chitosan degradation	Adjustable DD to suit different adhesives: High DD (>80%) chitosan, with more amino groups, suitable for cross-linking with aldehydes to prepare high-strength wood adhesives	[49,56]
Green Extraction Technologies	Enzymatic Hydrolysis (Deproteinization/Demineralization)	Enzyme type: Protease (alkaline/neutral protease), phosphatase; Enzyme activity: 1000–5000 U/g; Temperature: 30–50 °C; pH: 6–9; Time: 4–12 h	Environmentally friendly, non-corrosive, protein removal rate > 85%, minimal damage to chitin molecular chains, but enzyme cost is high (approximately 2–3 times that of acid/alkali method) and reaction cycle is long	Suitable for high-purity adhesive raw materials (e.g., medical/dental adhesives), avoiding chemical residue affecting biocompatibility	[57,58,59]
	Microbial Fermentation (Full-process Extraction)	Strain: Bacillus/Yeast; Fermentation temperature: 25–37 °C; pH: 5–8; Fermentation time: 24–72 h; Carbon-to-nitrogen ratio: 10–20:1	Simultaneous demineralization and deproteinization, no chemical reagents required, high product purity (chitin purity > 90%), but fermentation cycle is long and yield is low	Suitable for laboratory-scale small-batch preparation of high-purity chitosan for antibacterial adhesives (e.g., composite adhesives inhibiting wood mold)	[60,61]
	Ultrasound/Microwave-Assisted Extraction (Enhanced Demineralization/Deproteinization)	Ultrasound power: 200–500 W; Microwave power: 300–800 W; Assistance time: 10–60 min; Combined with acid/alkali method	Reduces traditional process time by 30–60%, lowers acid/alkali consumption by 20–40%, but requires specialized equipment and has high scale-up costs	Adaptable for upgrading existing industrial acid/alkali methods, improving production efficiency of wood adhesive raw materials and reducing energy consumption	[62,63,64,65]
Purification & Refining Processes	Impurity Removal (Decolorization/Desalting)	Decolorizing agent: Activated carbon/Hydrogen peroxide; Concentration: 1–5%; Temperature: 40–60 °C; Desalting: Deionized water dialysis/Ion exchange resin	Decolorization rate > 80%, ash content reduced to <1%, but activated carbon may adsorb some chitosan, reducing recovery yield	Used for high-transparency adhesives (e.g., packaging adhesives) or medical adhesives, avoiding impurities affecting appearance and biosafety	[66]
	Degree of Deacetylation (DD) Control	Stepwise deacetylation: Multiple treatments with low-concentration alkali (10–20% NaOH); Temperature gradient: 60 → 80 → 100 °C; Time: 1–3 h/step	DD precision control ±2%, enabling preparation of chitosan with specific DD (e.g., 60%, 80%) to meet different cross-linking requirements	High DD (>80%) suitable for aldehyde cross-linking (e.g., dialdehyde starch); low DD (50–60%) suitable for acidic cross-linking (e.g., citric acid); used for wood/paper adhesives	[67,68]
	Molecular Weight Control (Degradation/Fractionation)	Degradation: Hydrogen peroxide (concentration 0.5–2%)/Ultrasound (300–600 W); Fractionation: Ethanol precipitation (concentration 30–70%)	Molecular weight adjustable from 1 × 10^4^ to 1 × 10^6^ Da, narrow distribution (PDI 1.2–1.8), but degradation may introduce oxygen-containing impurities	Low molecular weight (<1 × 10^5^ Da) chitosan suitable for low-viscosity coating adhesives (e.g., plywood); high molecular weight (>5 × 10^5^ Da) used for high-strength structural adhesives	[69]

**Table 3:** Performance testing system for chitin adhesives.

Adhesive Type	Curing Temperature	Curing Time	Curing Pressure	Test Standard	Reference
CS-PAA@Ca^2+^	120 °C	6.5 min	1 MPa	Industry-standard severe “4+4+1” test: boiling water immersion for 4 h → drying for 20 h → boiling water re-immersion for 4 h → cold water immersion for 1 h, followed by testing	[86]
Hemicellulose–Chitosan Composite Adhesive	120 °C	2.5 min	1.67 MPa	European Standards EN 204, EN 205	[87]
SM/DCS Composite Adhesive	120 °C	6 min	1 MPa	No specific standard; experimental conditions: 30 °C, 99% relative humidity (RH), continued for 30 d, observing mold growth	[13]
CSC-G Carboxylated Chitosan–Glucose Adhesive	140–200 °C (optimum 160 °C)	1–10 min (optimum 3 min)	1 MPa	Chinese Standards GB/T 9846-2015, GB/T 17657-2013	[72]
Chitosan–Dopamine Composite Adhesive	Room temperature—120 °C	2 h	Adjusted as needed (no explicit pressure specified in some cases)	No specific standard; experimental conditions applied	[88]

**Table 4:** Different Extraction Methods and Their Characteristics.

Key Parameter	Influence Pattern on Bonding Performance	Specific Manifestations	References
Degree of Deacetylation (DD)	Positive correlation dominates; higher DD favors improved bonding strength and water resistance.	When DD ≥ 90%, wet shear strength is significantly increased (e.g., reaching 1.40 MPa for CS-PB adhesive). Increased amino group (-NH_2_) content enhances hydrogen bonding/electrostatic interactions with wood hydroxyl groups and crosslinking reactivity. Excessively high DD (>95%) may reduce solubility due to increased crystallinity, slightly weakening interfacial bonding.	[9,89,90]
Molecular Weight (Mw)	Medium molecular weight (50,000–300,000 Da) is optimal; too low or too high is detrimental.	Low Mw (<50,000 Da): High penetration but weak cohesion, dry shear strength < 1.5 MPa. Medium Mw: Balances penetration and cohesion, dry shear strength can reach 2.3–5.6 MPa. High Mw (>500,000 Da): High viscosity, poor penetration, prone to forming “starved glue lines,” resulting in decreased wet strength.	[71]
Crosslinking Density	Moderate crosslinking is optimal; either too high or too low impairs performance.	Low crosslinking density: Loose network, poor water resistance (wet strength < 0.5 MPa). Moderate crosslinking (e.g., GL/C = 1:1): Forms a dense three-dimensional network, achieving dry strength > 2.0 MPa and wet strength > 0.8 MPa. Excessively high crosslinking density: Increased brittleness, stress concentration at the interface, leading to decreased bonding strength.	[21,90]

**Table 5:** Reagents Used in Graft Polymerization and Their Functions.

Reagent Name	Reagent Type	Function Description	Reference
**Sodium Tripolyphosphate (TPP)**	Ionic Crosslinking Agent	Interacts electrostatically with the amino groups of chitosan to form ionic crosslinks, facilitating the preparation of nanoparticles and enhancing their stability and dispersibility.	[96]
**Glutaraldehyde**	Covalent Crosslinking Agent	Undergoes a Schiff base reaction with chitosan amino groups to form imine crosslinks, constructing a porous network structure that enhances adsorption capacity and thermal stability.	[30]
**Citric Acid (CA)**	Crosslinker/Solvent	Dissolves chitosan and facilitates supramolecular self-assembly via ionic and hydrogen bonding. After hot-pressing, ester and amide bonds form, enabling viscosity modulation and improving water resistance.	[9]
**Glycerol Triglycidyl Ether (GTE)**	Epoxy Modifier	Epoxidizes lignosulfonate by introducing epoxy groups, enhancing crosslinking reactions with amino and hydroxyl groups of chitosan, and constructing a dense three-dimensional network.	[21]
**1,4-Phenylenediboronic Acid (PBA)**	Dynamic Covalent Crosslinker	Forms boronic ester dynamic bonds with lignin-derived 3,4-dihydroxybenzaldehyde, which subsequently reacts with chitosan amino groups to form imine bonds, endowing the adhesive with recyclability and high bonding strength.	[90]
**Poly(sodium 4-styrenesulfonate) (PSS)**	Polyanionic Electrolyte	Forms polyelectrolyte complexes (PECs) with cationic chitosan via electrostatic interactions, stabilizing the system. Simultaneously, π-π stacking occurs with lignin, improving compatibility.	[9]
**3,4-Dihydroxybenzaldehyde (DBA)**	Functional Monomer/Crosslinking Intermediate	Reacts with 1,4-phenylenediboronic acid to generate a dialdehyde-containing monomer, which then forms imine bonds with chitosan, constructing a dynamic crosslinked network that enhances bonding strength and mildew resistance.	[90]

## Data Availability

The data supporting the conclusions are included in the main manuscript.
